# Supplementary material for: Novel and Potent Acetylcholinesterase Inhibitors for the Treatment of Alzheimer’s Disease from Natural (±)-7,8-Dihydroxy-3-methyl-isochroman-4-one
Source: Molecules. 2022 May 11;27(10):3090. doi: 10.3390/molecules27103090 (PMC9145193; doi:10.3390/molecules27103090)

Article

Novel and Potent Acetylcholinesterase Inhibitors for the Treatment of Alzheimer's Disease from Natural (±)-7,8-Dihydroxy-3-methyl-isochroman-4-one

Xinnan Li [1], Yilin Jia [1], Junda Li [1], Pengfei Zhang [1], Tiantian Li [2], Li Lu [2], Hequan Yao [1,*], Jie Liu [3,*], Zheying Zhu [2] and Jinyi Xu [1,*]

[1] State Key Laboratory of Natural Medicines, Department of Medicinal Chemistry, China Pharmaceutical University, #639 Longmian Avenue, Jiangning District, Nanjing 211198, China; xinnanli@126.com (X.L.); ammie10@163.com (Y.J.); zgykdxljd0938@163.com (J.L.); zpf834220218@163.com (P.Z.)

[2] Division of Molecular Therapeutics & Formulation, School of Pharmacy, The University of Nottingham, University Park Campus, Nottingham NG7 2RD, UK; paytl4@nottingham.ac.uk (T.L.); Li.Lu@nottingham.ac.uk (L.L.); Zheying.Zhu@nottingham.ac.uk (Z.Z.)

[3] Department of Organic Chemistry, China Pharmaceutical University, Nanjing 211198, China

* Correspondence: hyao@cpu.edu.cn (H.Y.); cpu-jill@163.com (J.L.); jinyixu@china.com (J.X.)

^{1}H-NMR and ^{13}C-HMR spectra of compound **10a**

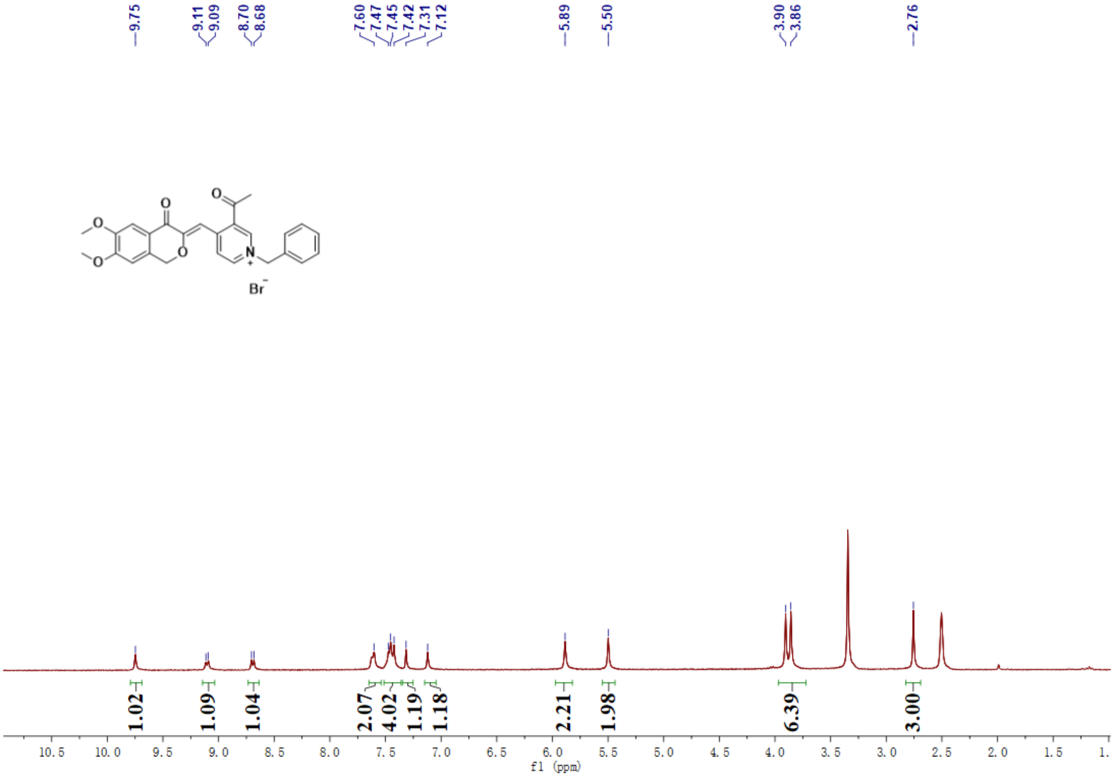

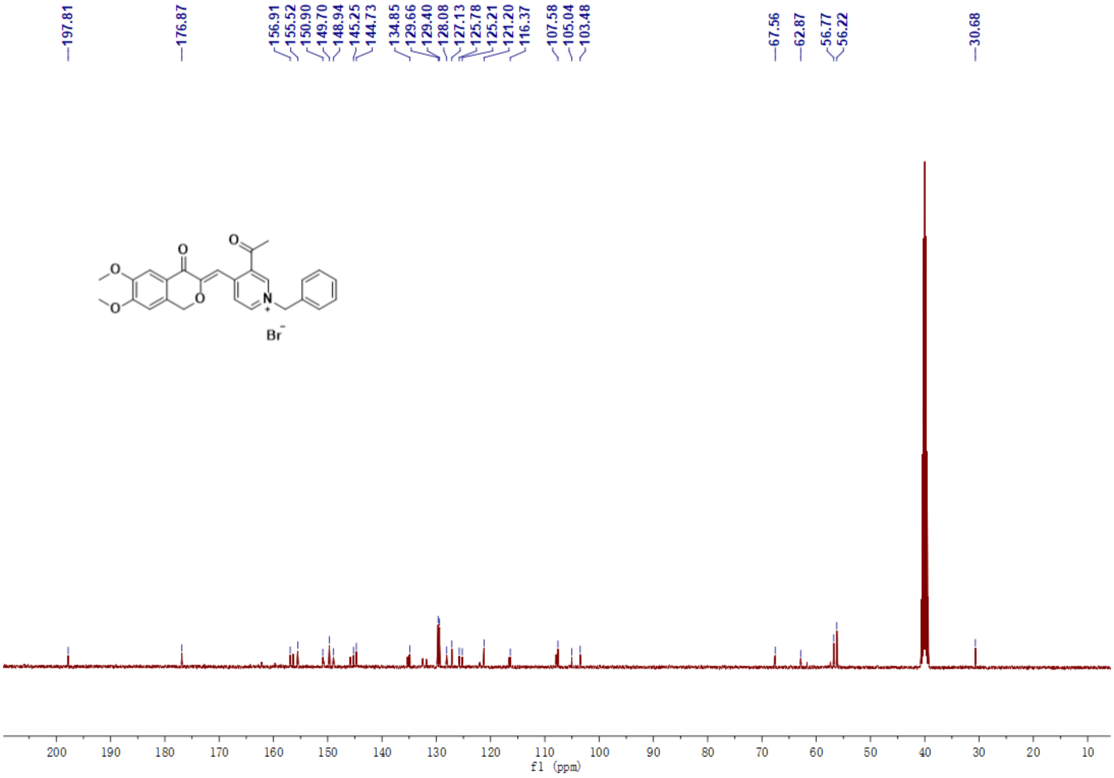

^{1}H-NMR and ^{13}C-HMR spectra of compound **10b**

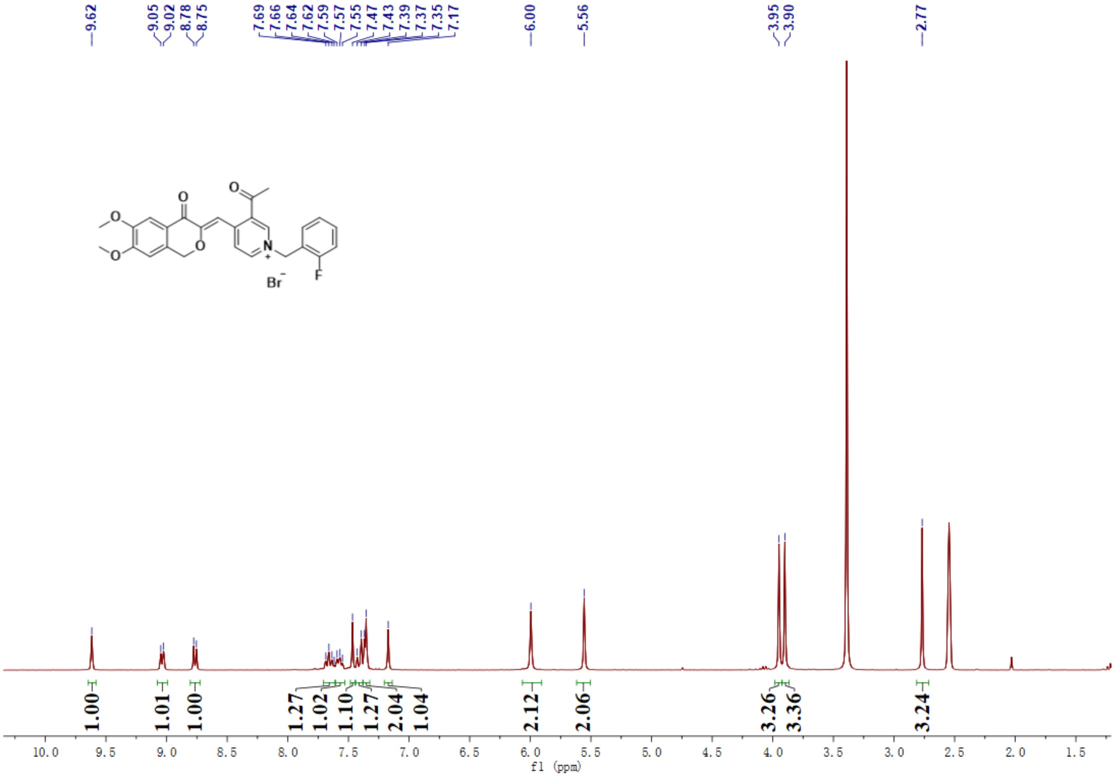

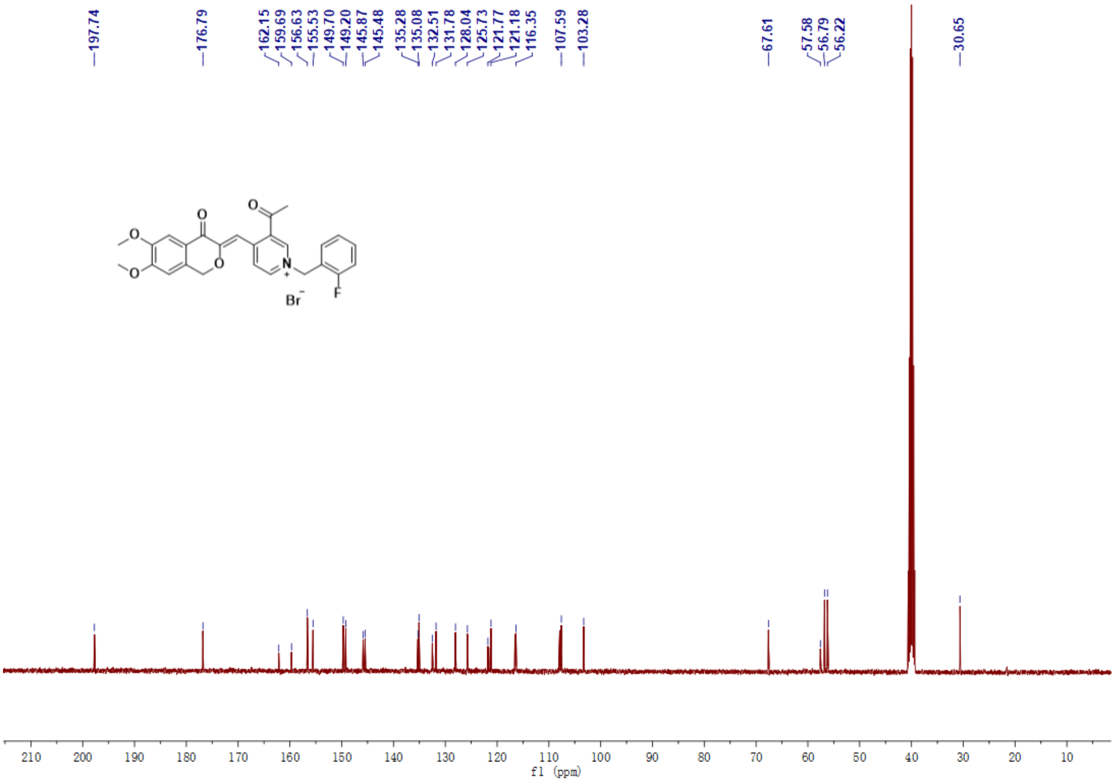

^{1}H-NMR and ^{13}C-HMR spectra of compound **10c**

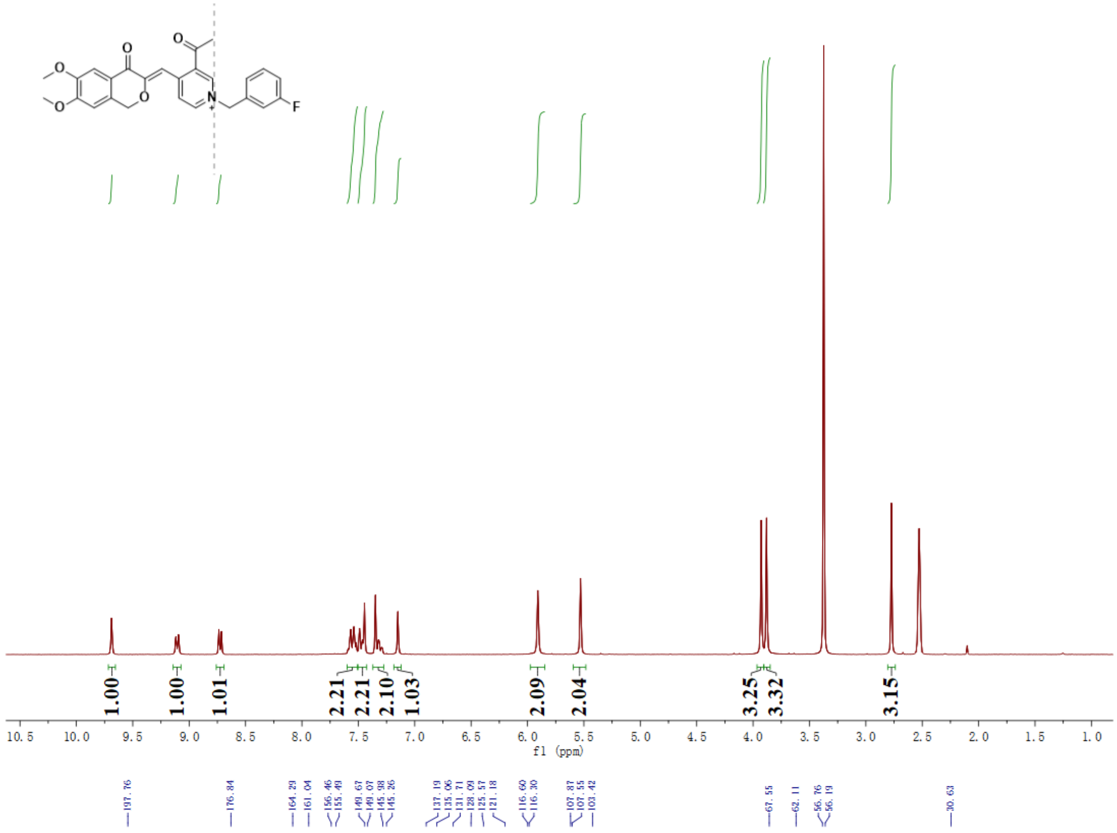

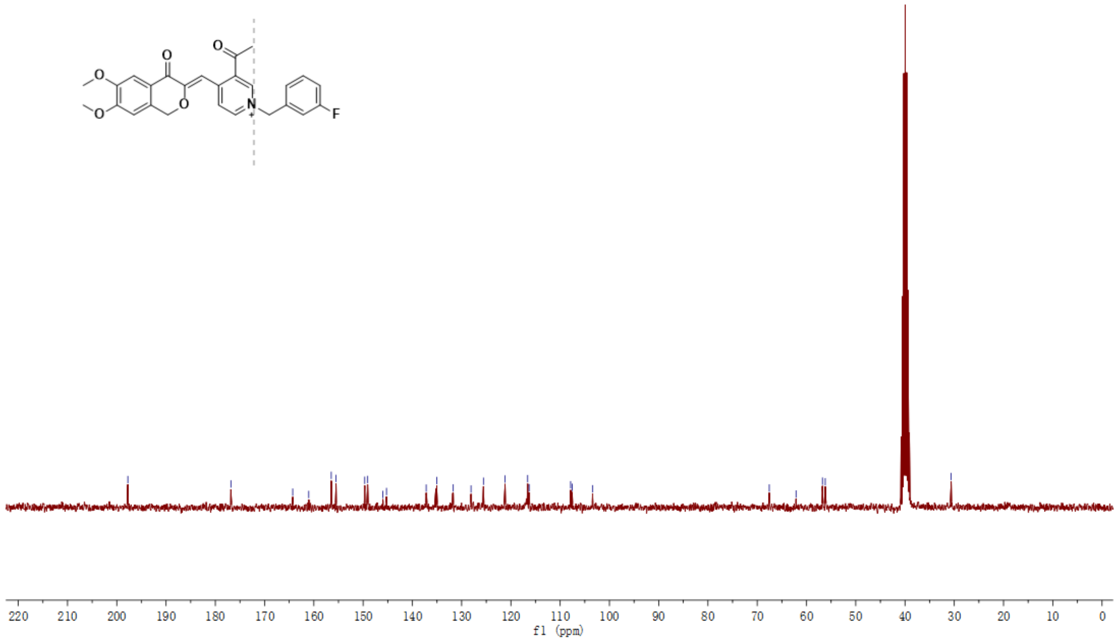

^{1}H-NMR and ^{13}C-HMR spectra of compound **10d**

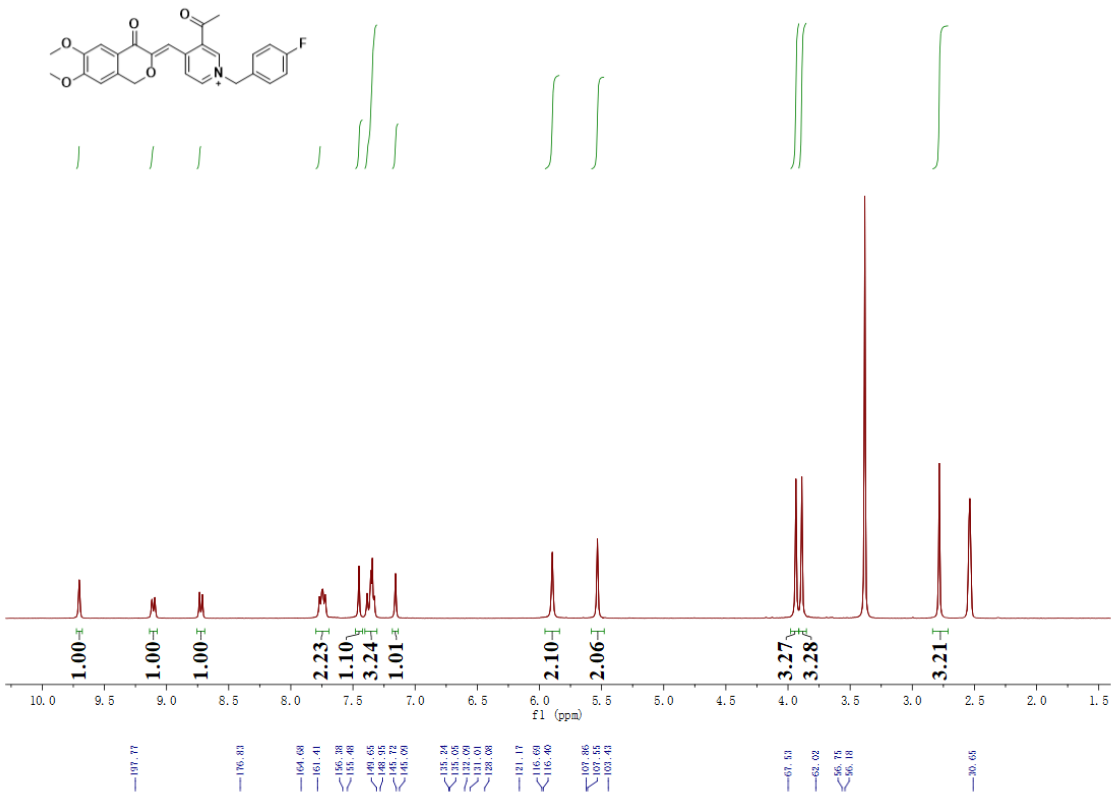

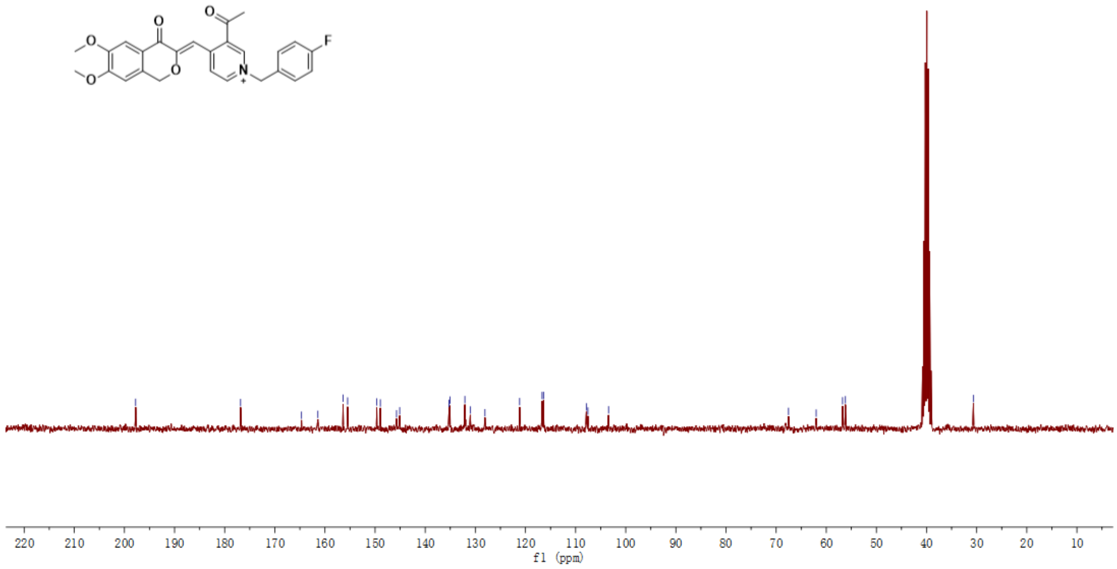

^{1}H-NMR and ^{13}C-HMR spectra of compound **10e**

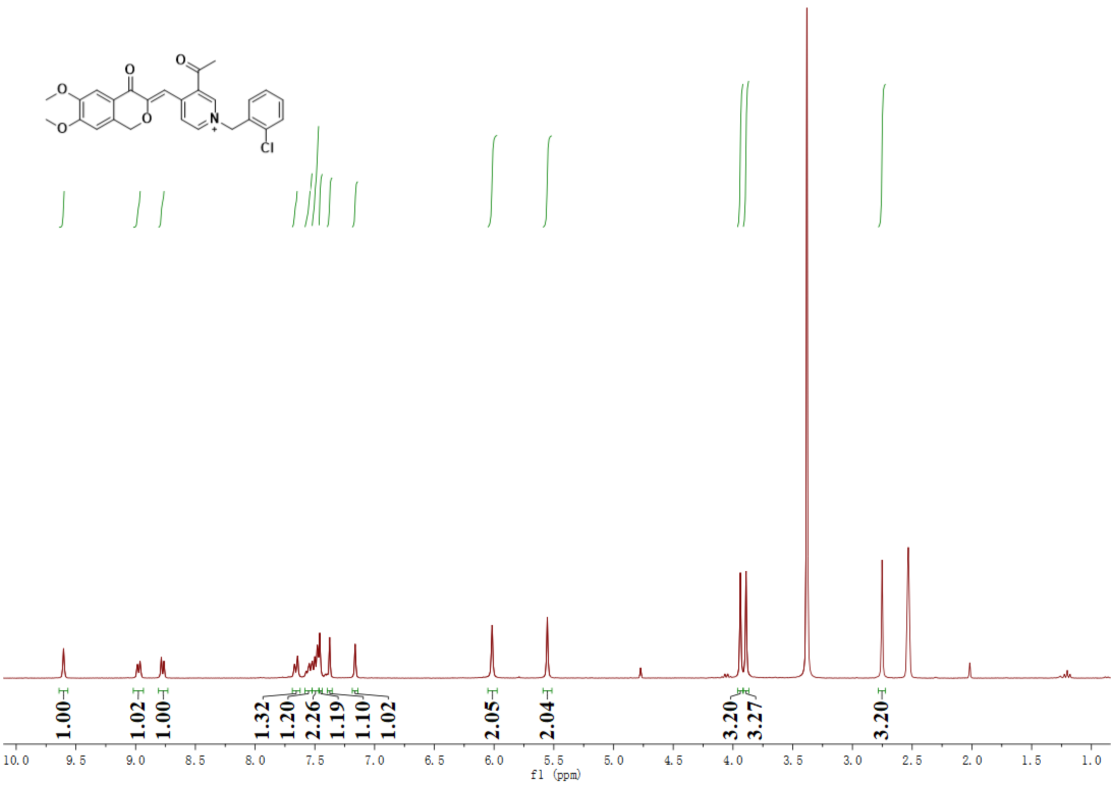

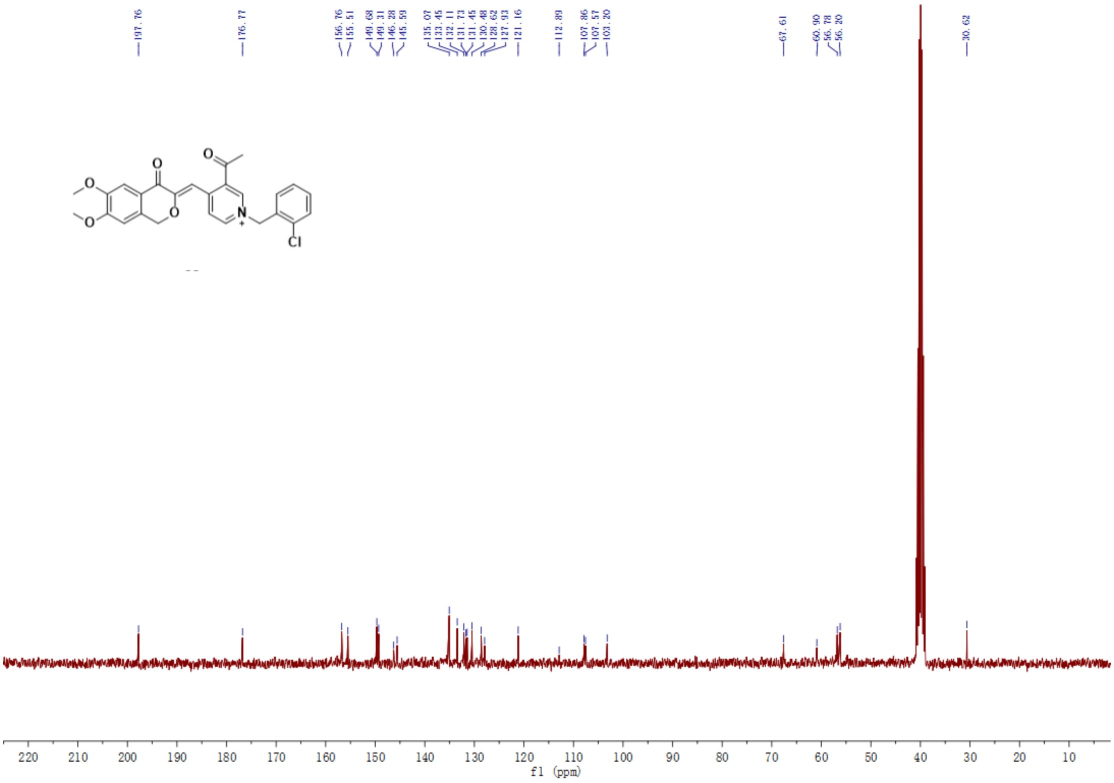

^{1}H-NMR and ^{13}C-HMR spectra of compound **10f**

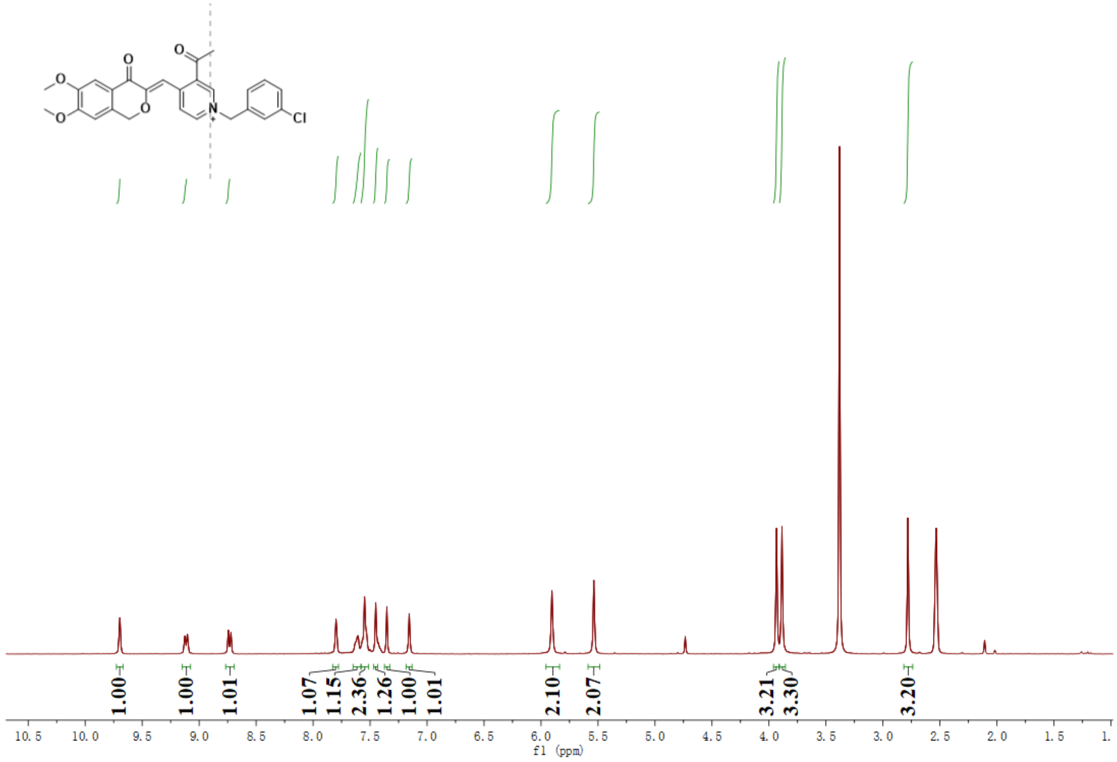

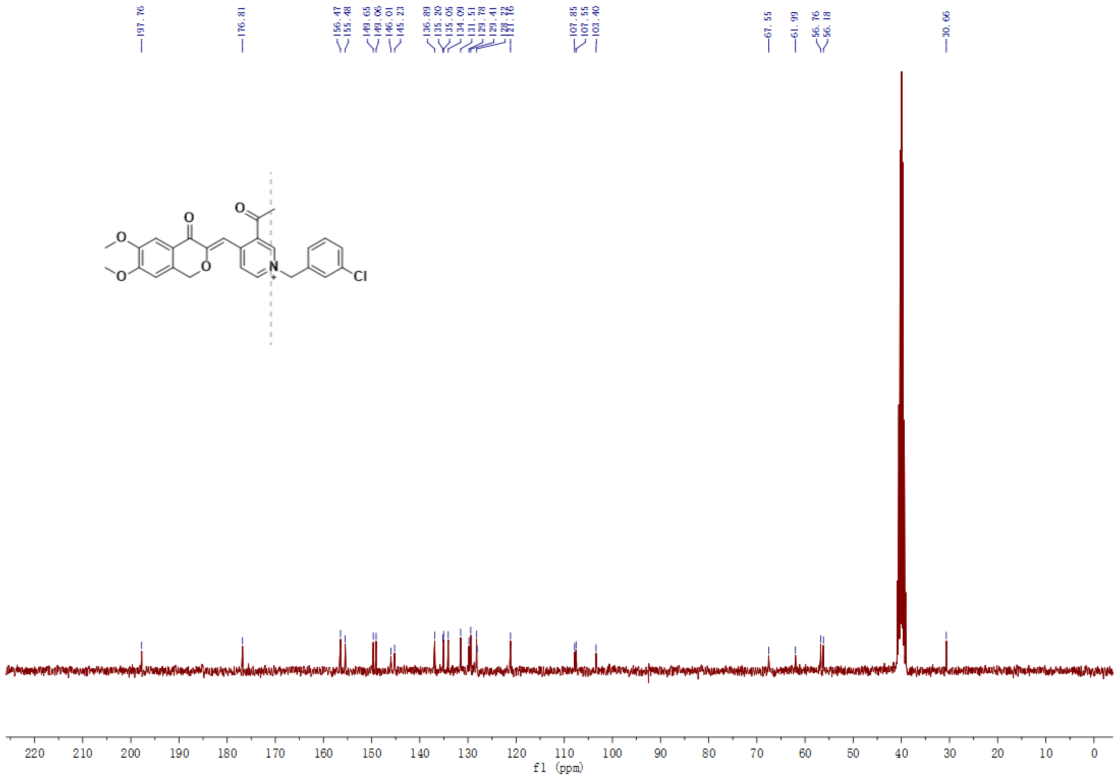

^{1}H-NMR and ^{13}C-HMR spectra of compound **10g**

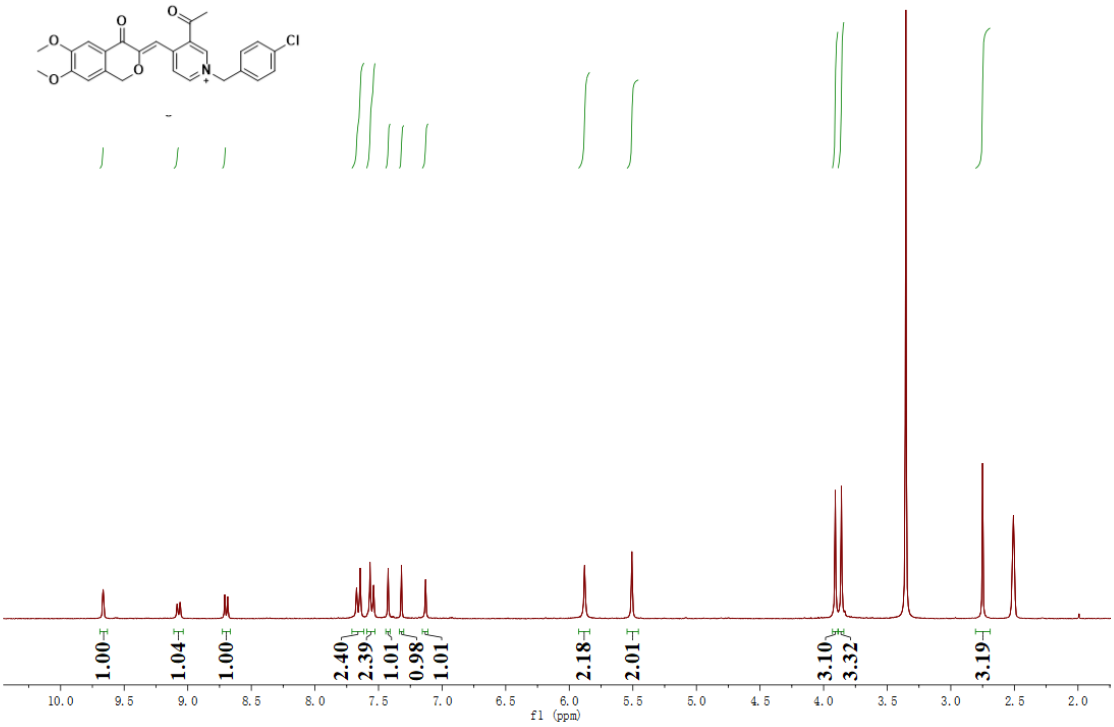

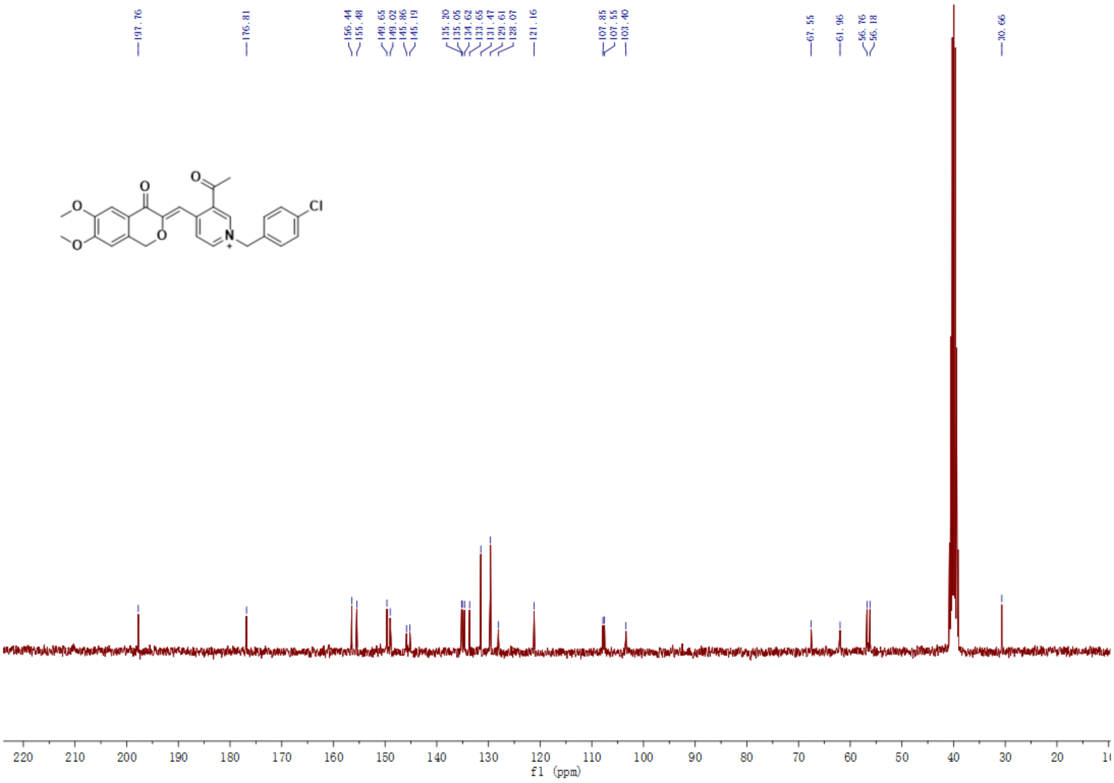

^{1}H-NMR and ^{13}C-HMR spectra of compound **10h**

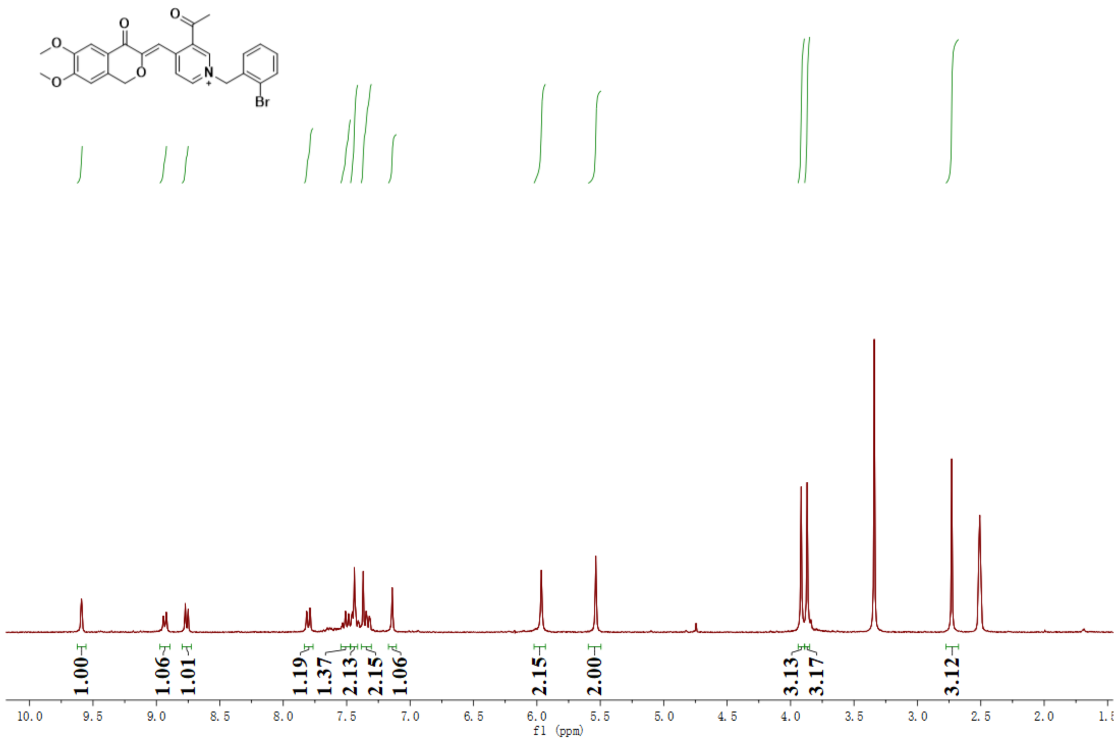

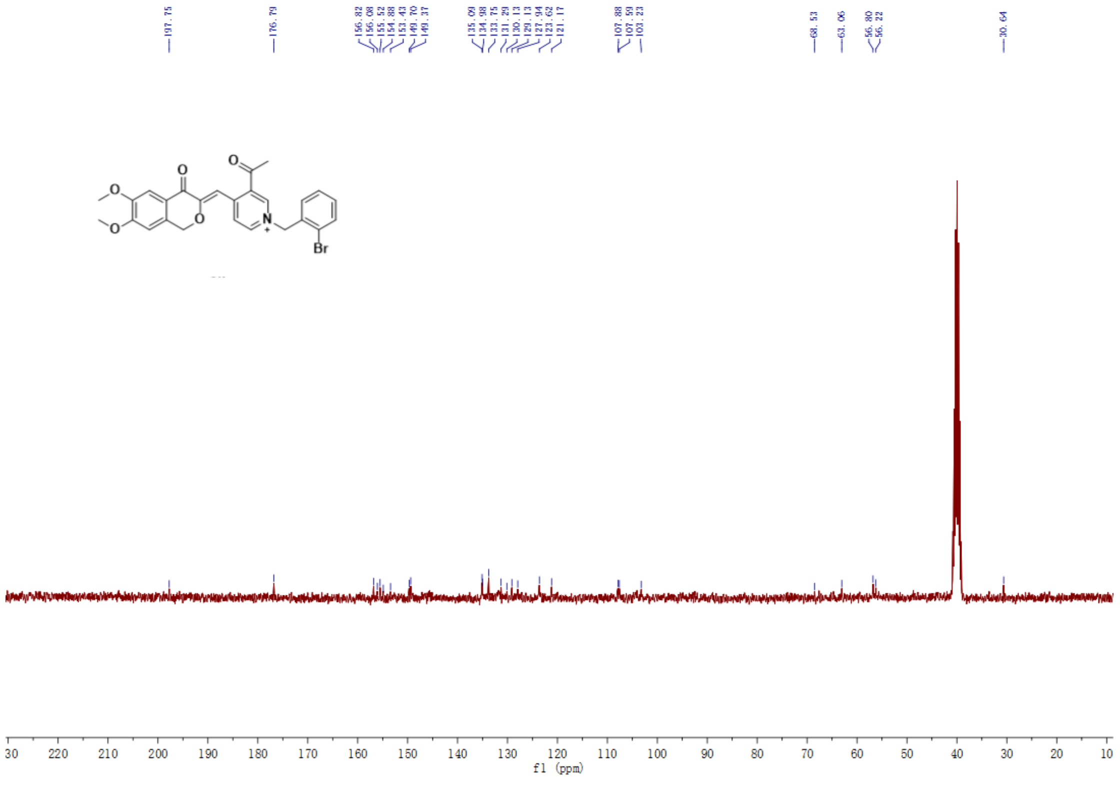

^{1}H-NMR and ^{13}C-HMR spectra of compound **10i**

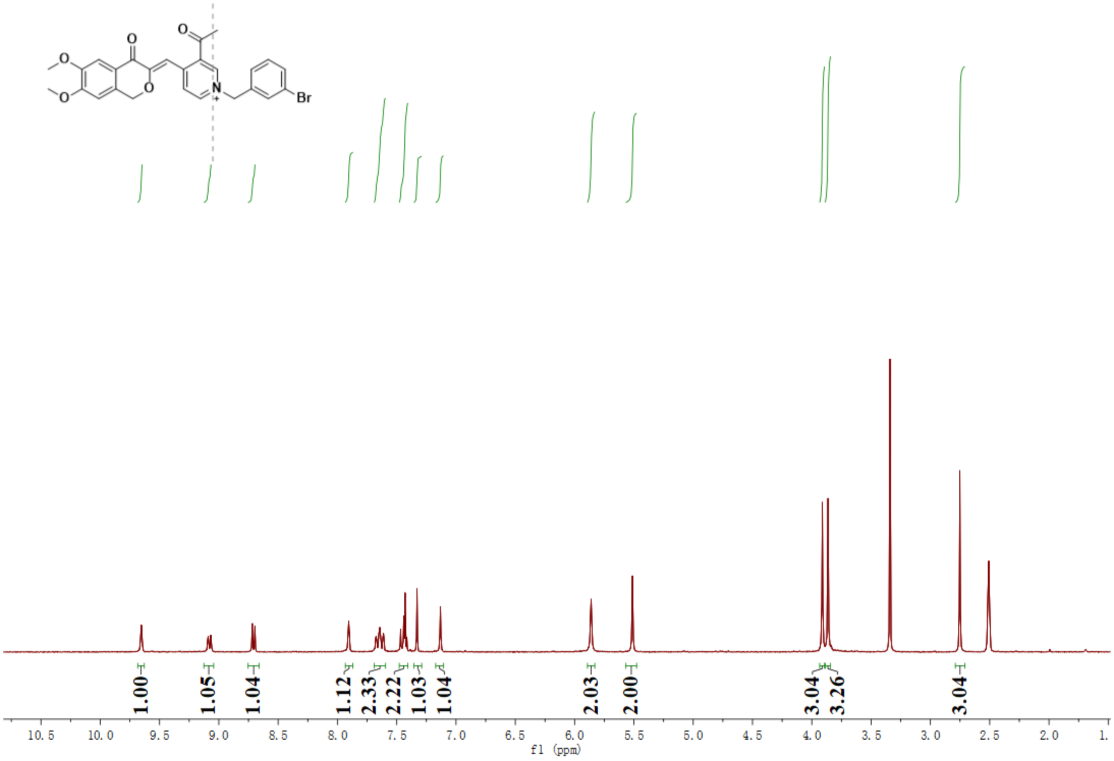

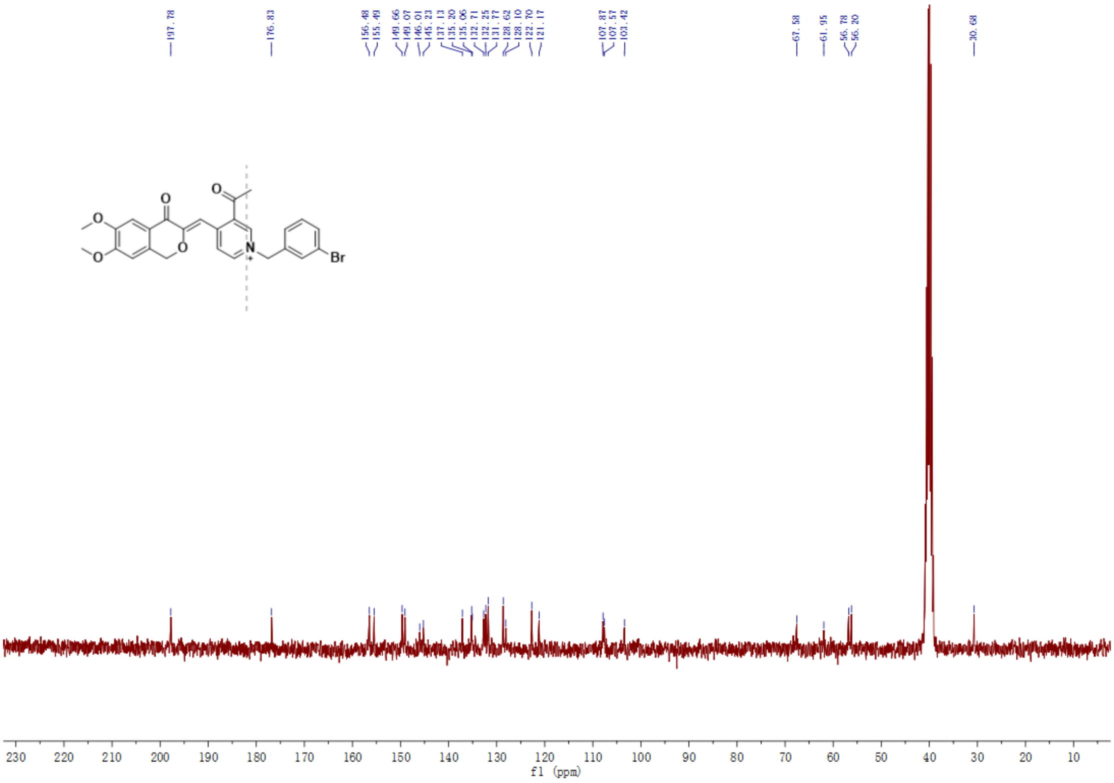

^{1}H-NMR and ^{13}C-HMR spectra of compound **10j**

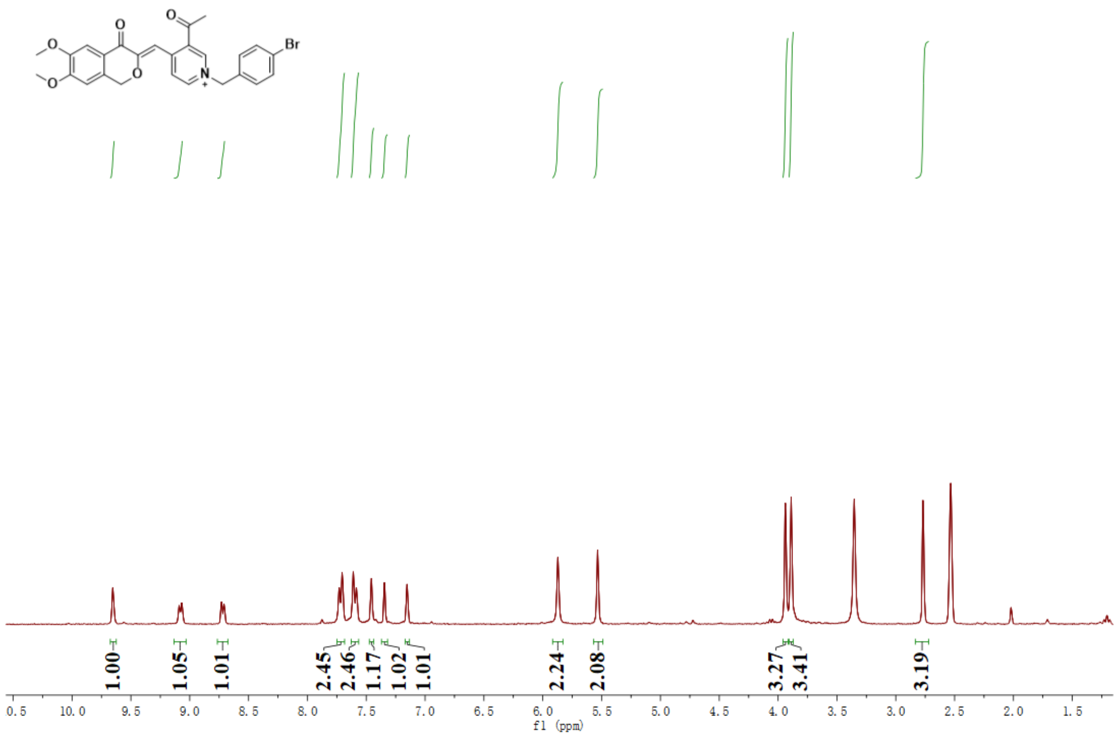

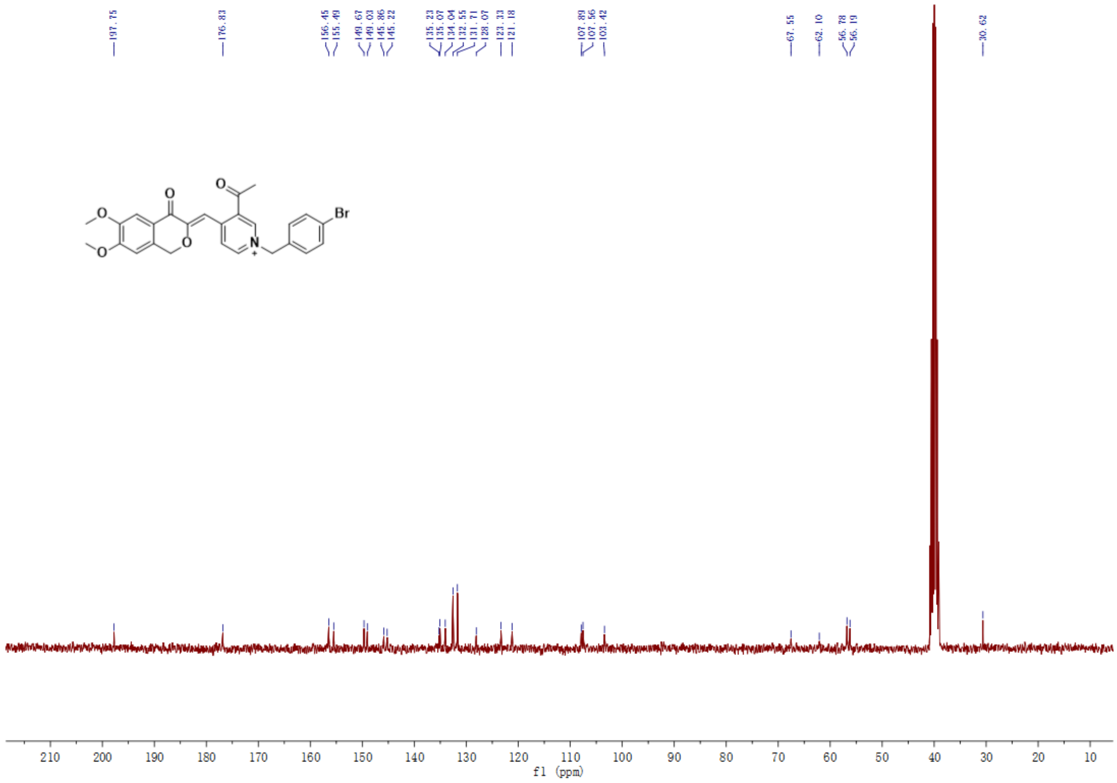

^{1}H-NMR and ^{13}C-HMR spectra of compound **10k**

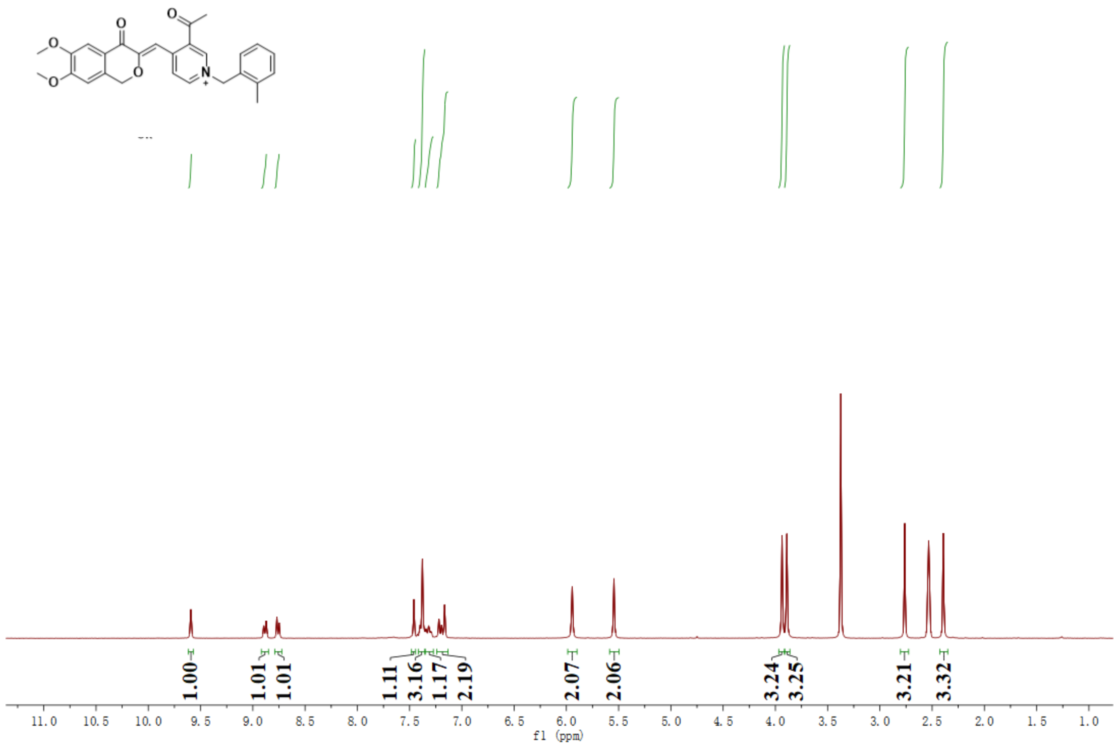

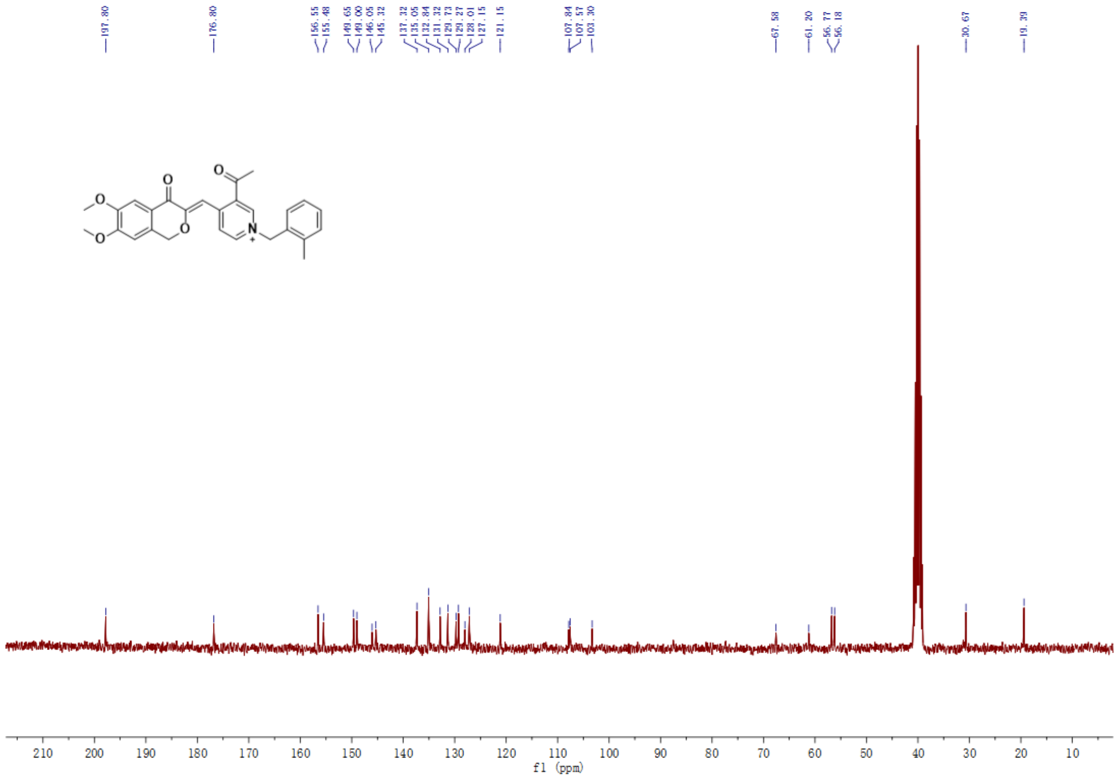

^{1}H-NMR and ^{13}C-HMR spectra of compound **10l**

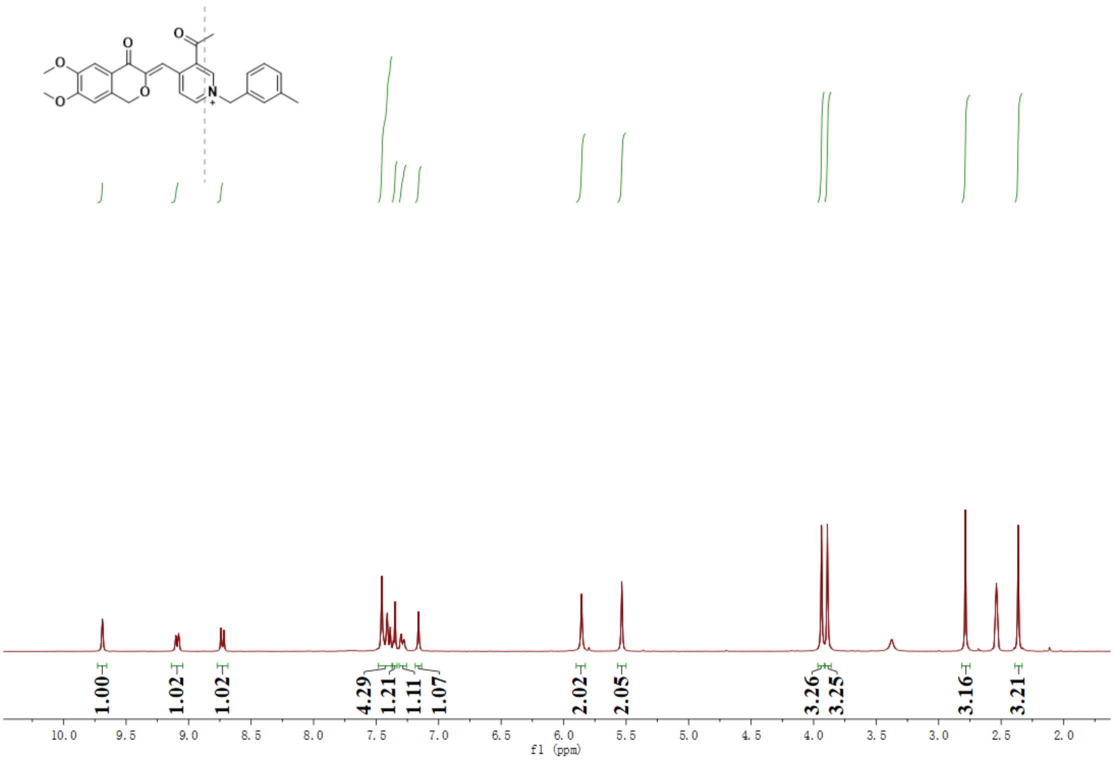

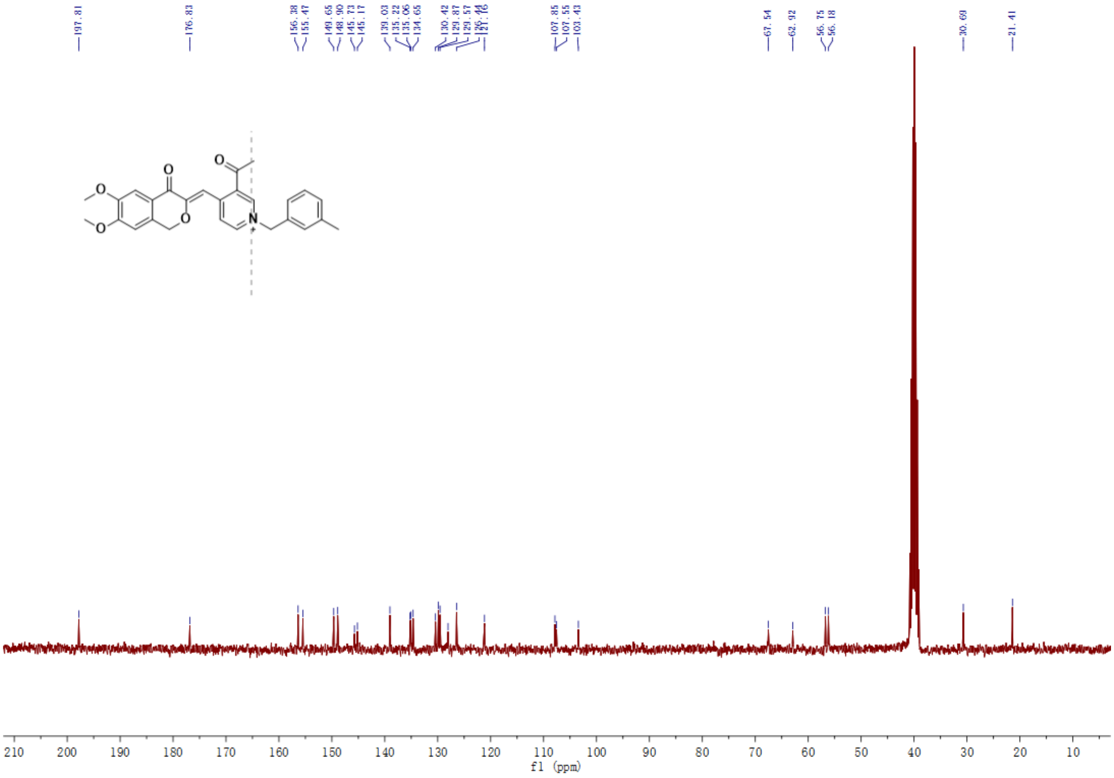

^{1}H-NMR and ^{13}C-HMR spectra of compound **10m**

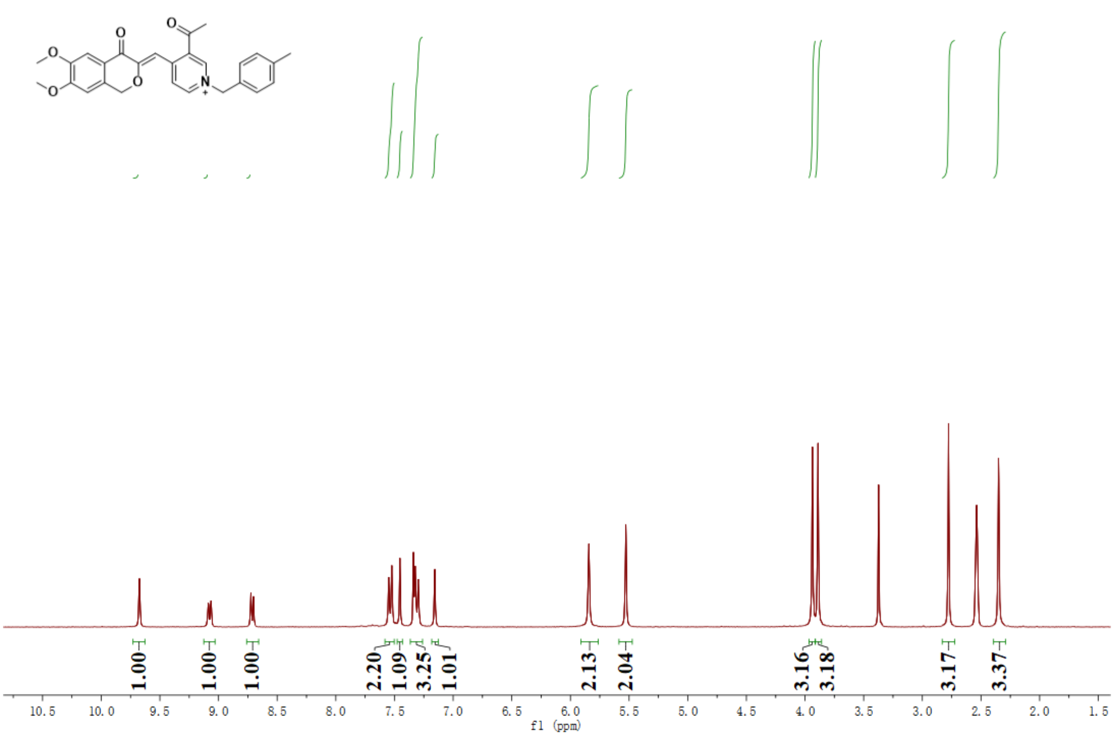

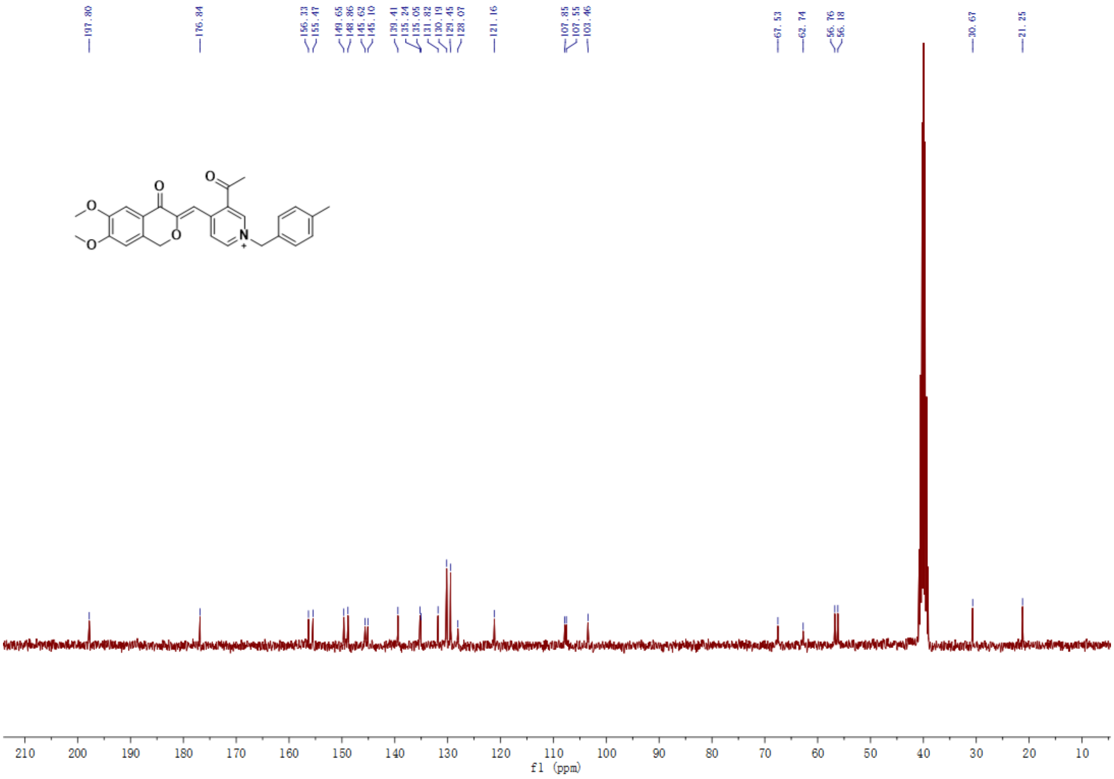

^{1}H-NMR and ^{13}C-HMR spectra of compound **10n**

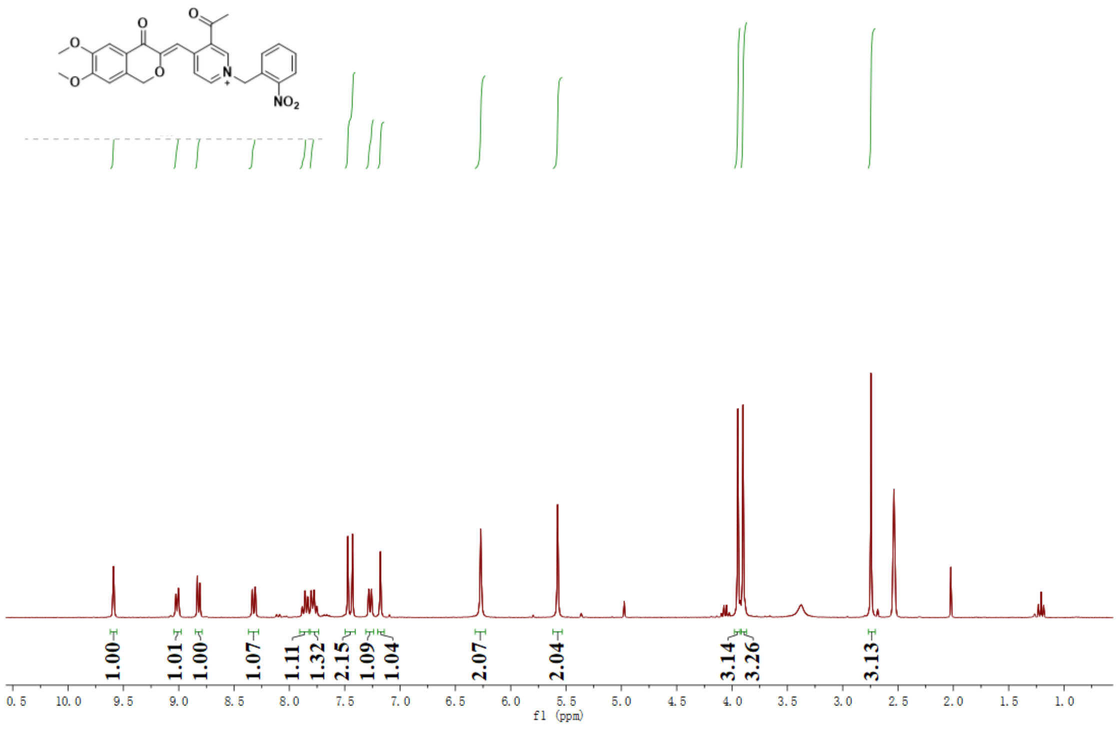

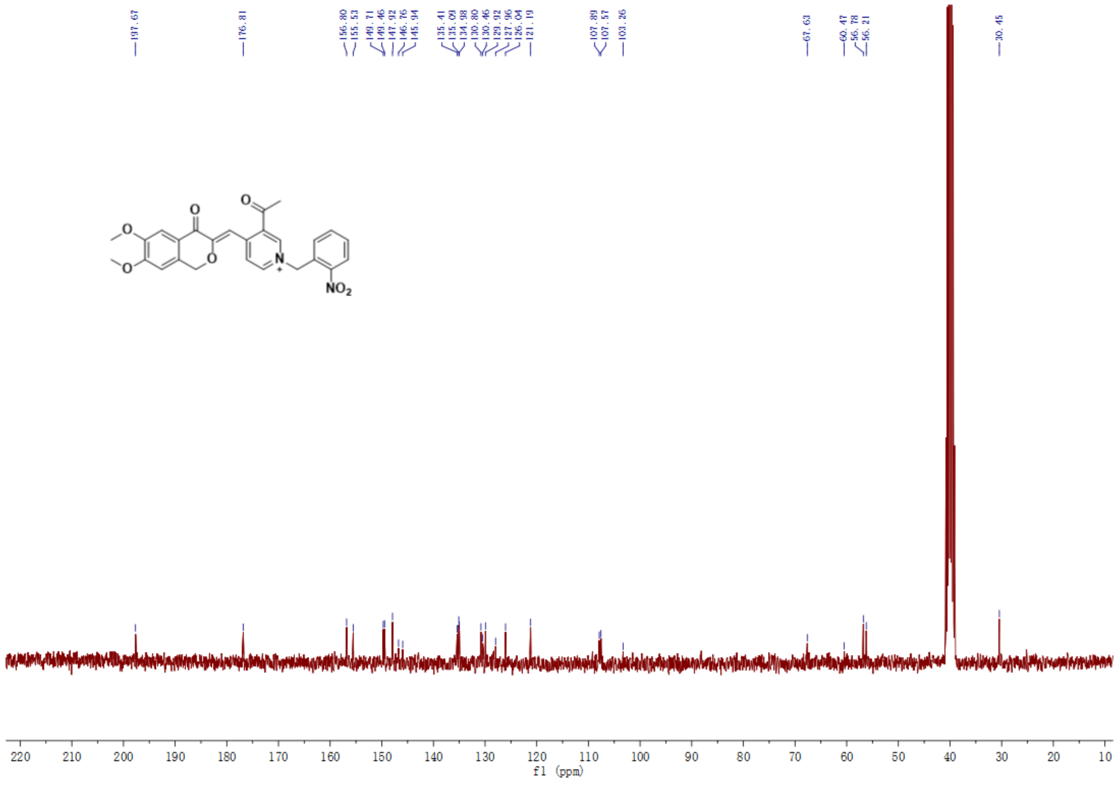

^{1}H-NMR and ^{13}C-HMR spectra of compound **10o**

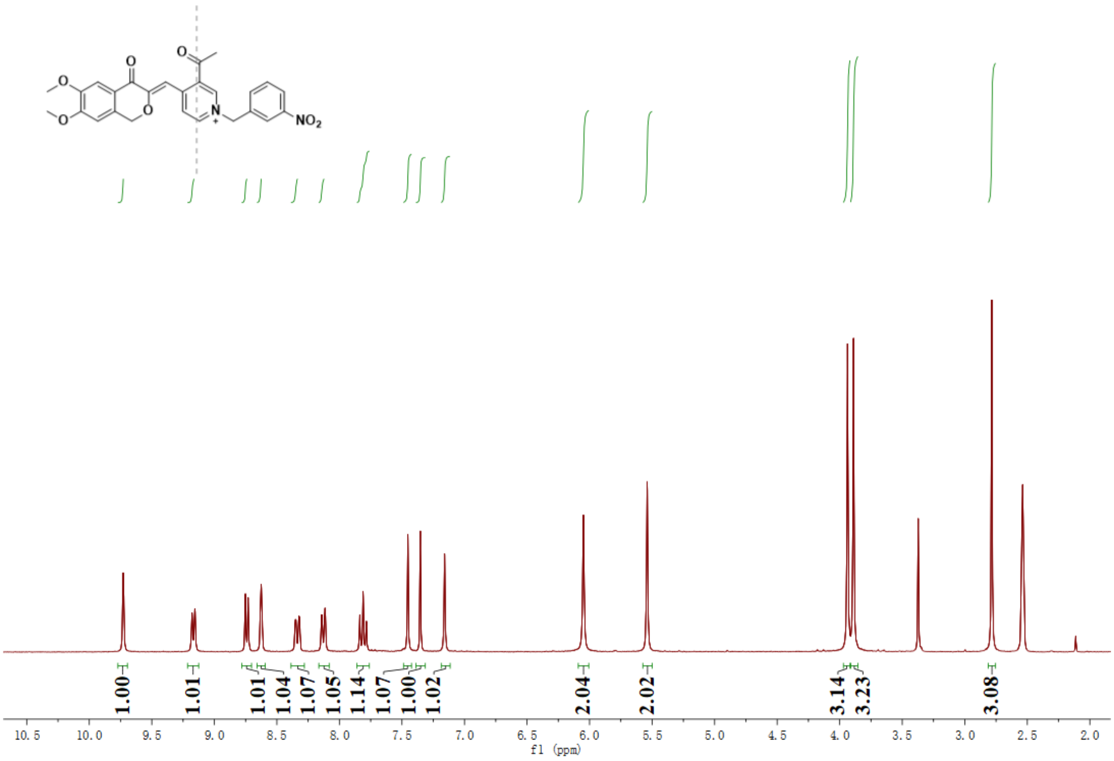

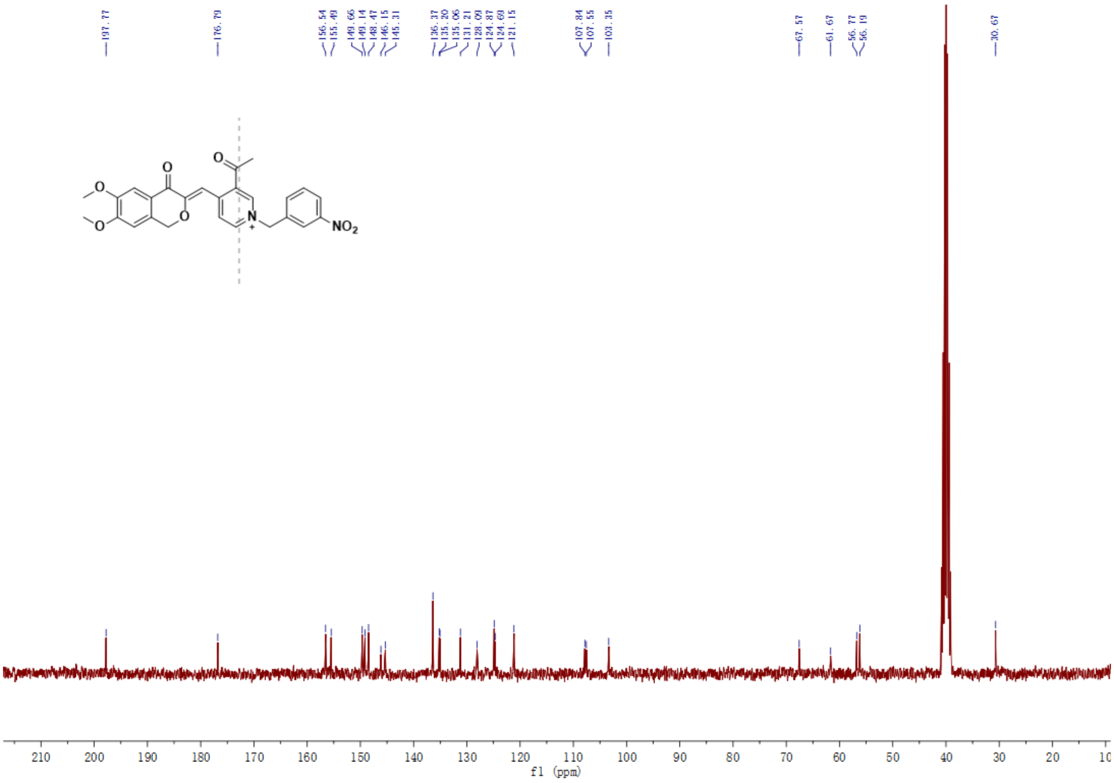

^{1}H-NMR and ^{13}C-HMR spectra of compound **10p**

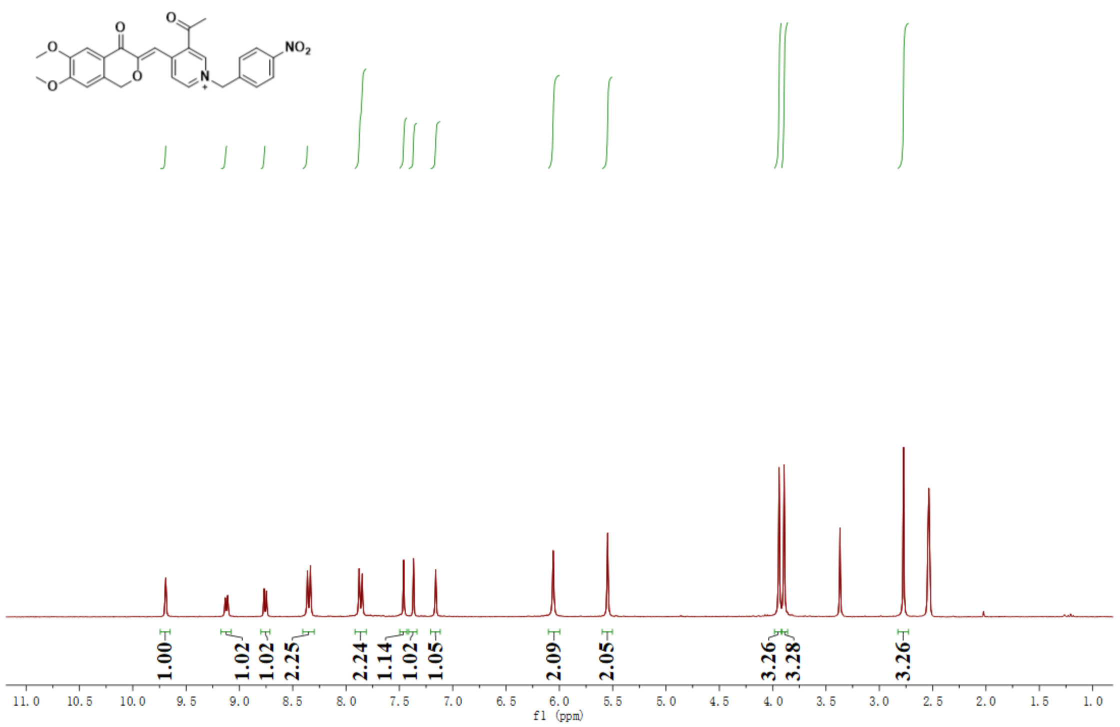

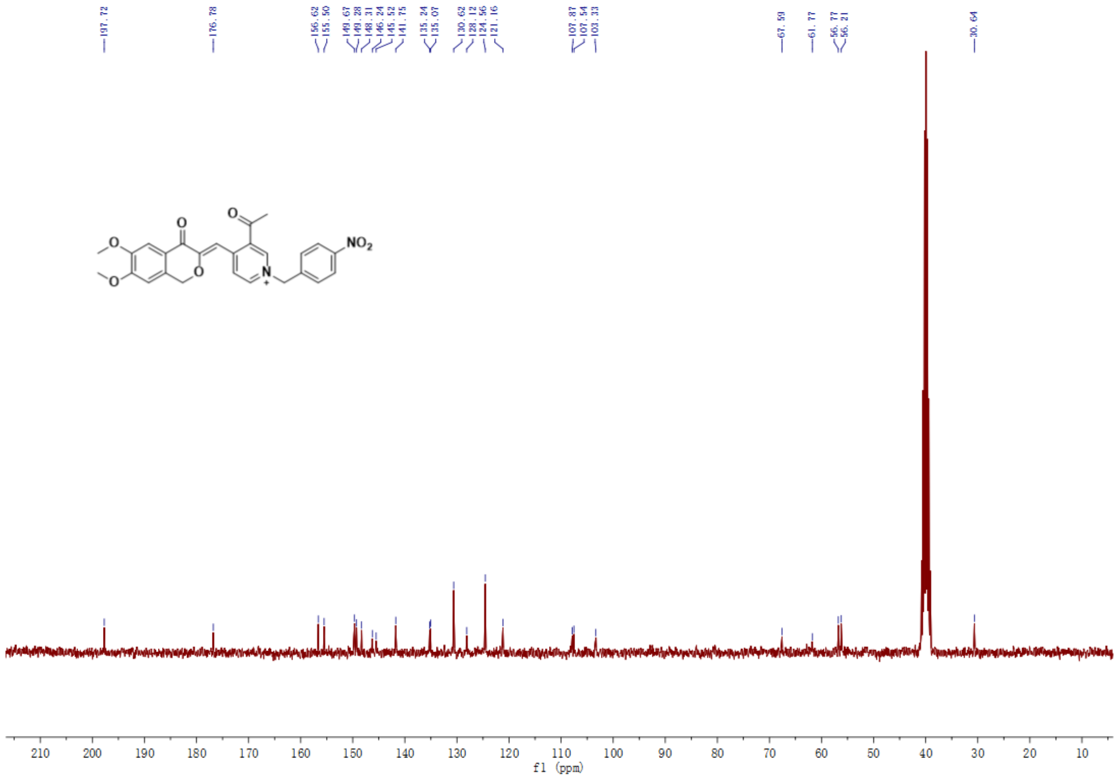

^{1}H-NMR and ^{13}C-HMR spectra of compound **10q**

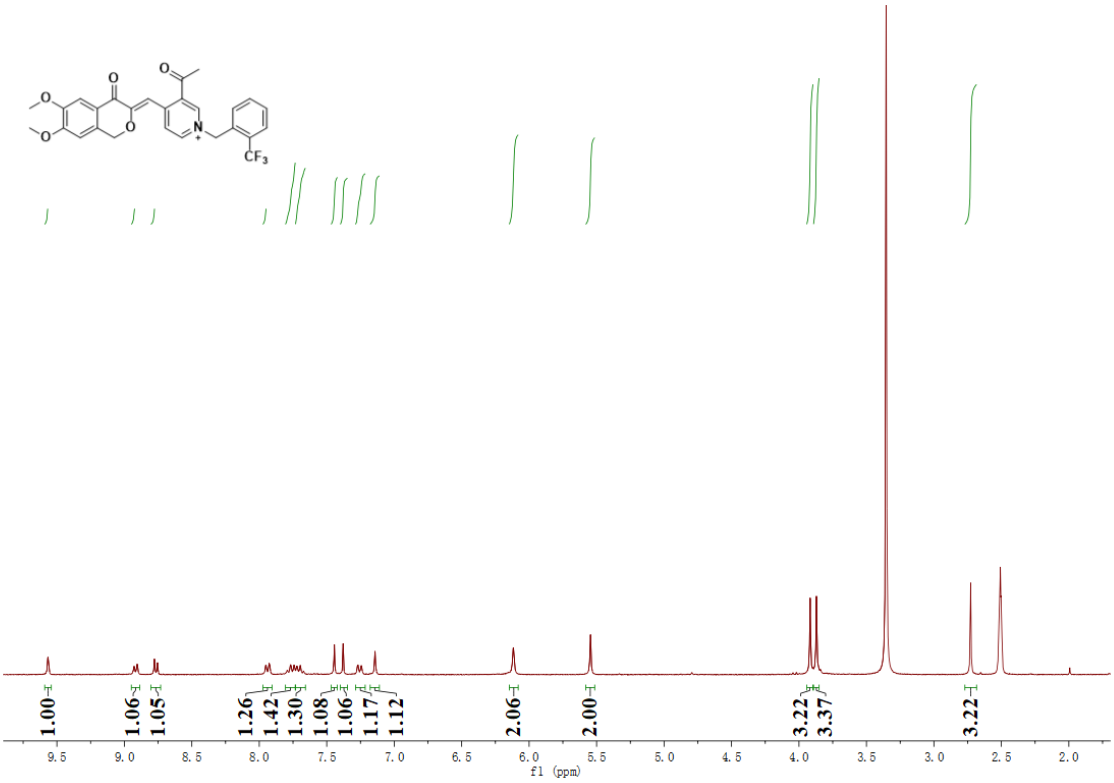

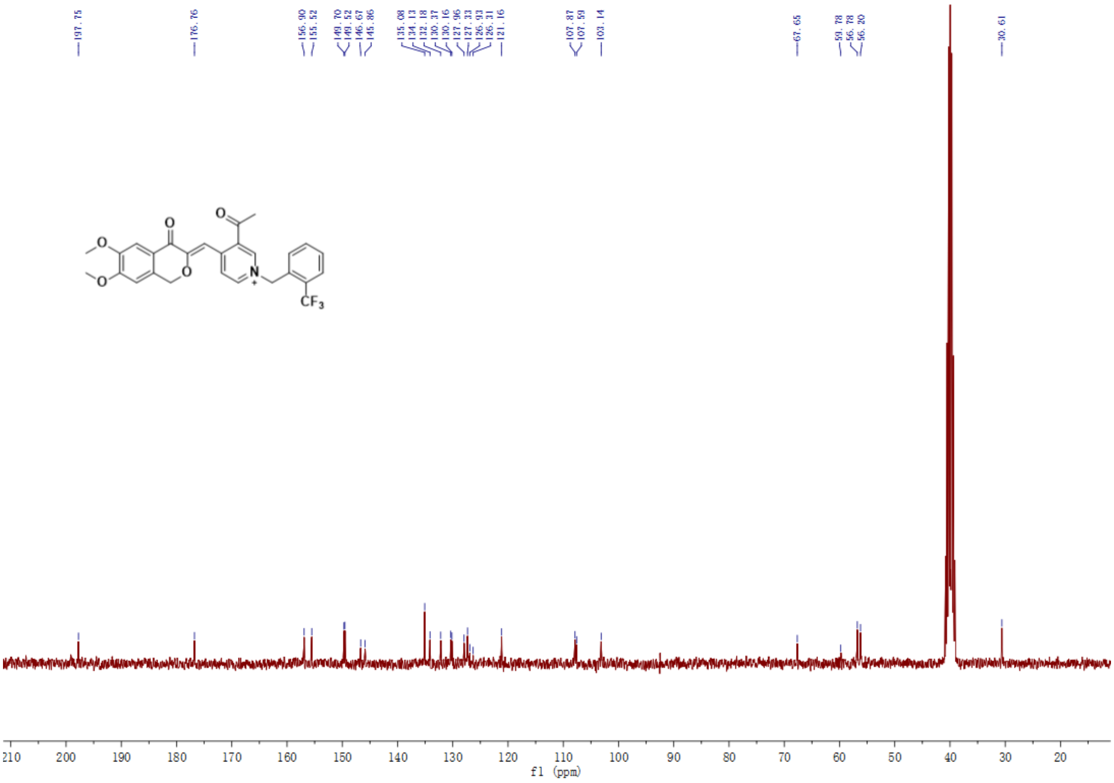

^{1}H-NMR and ^{13}C-HMR spectra of compound **10r**

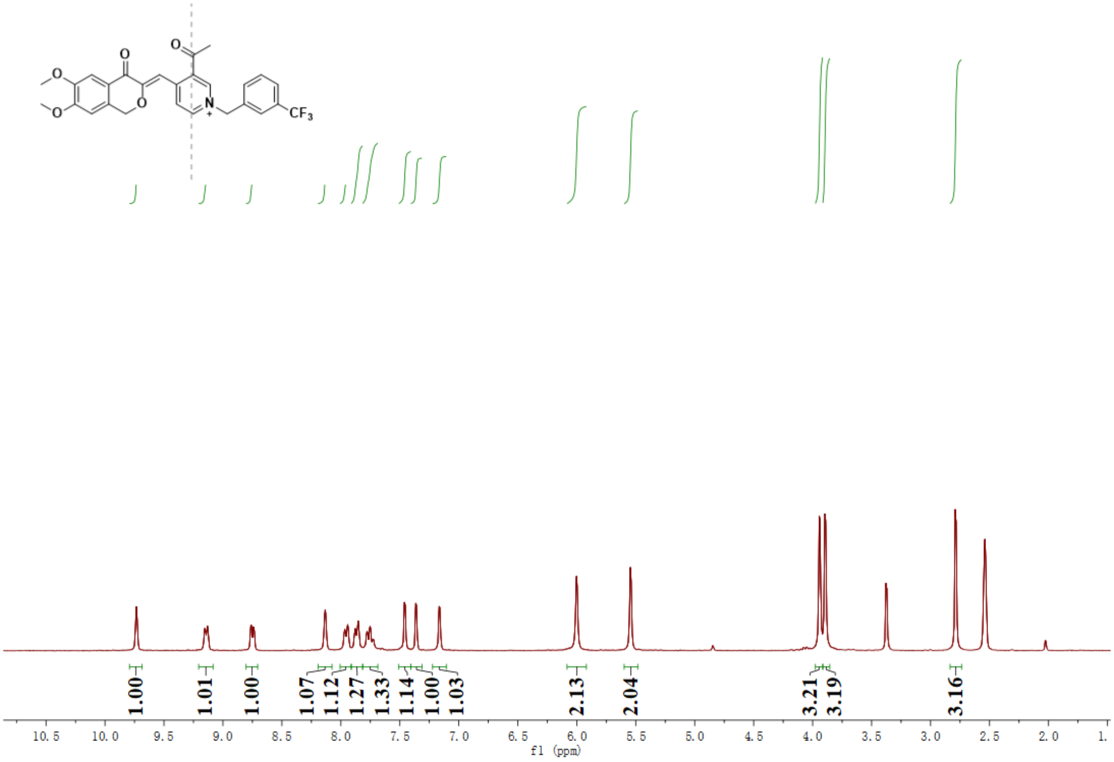

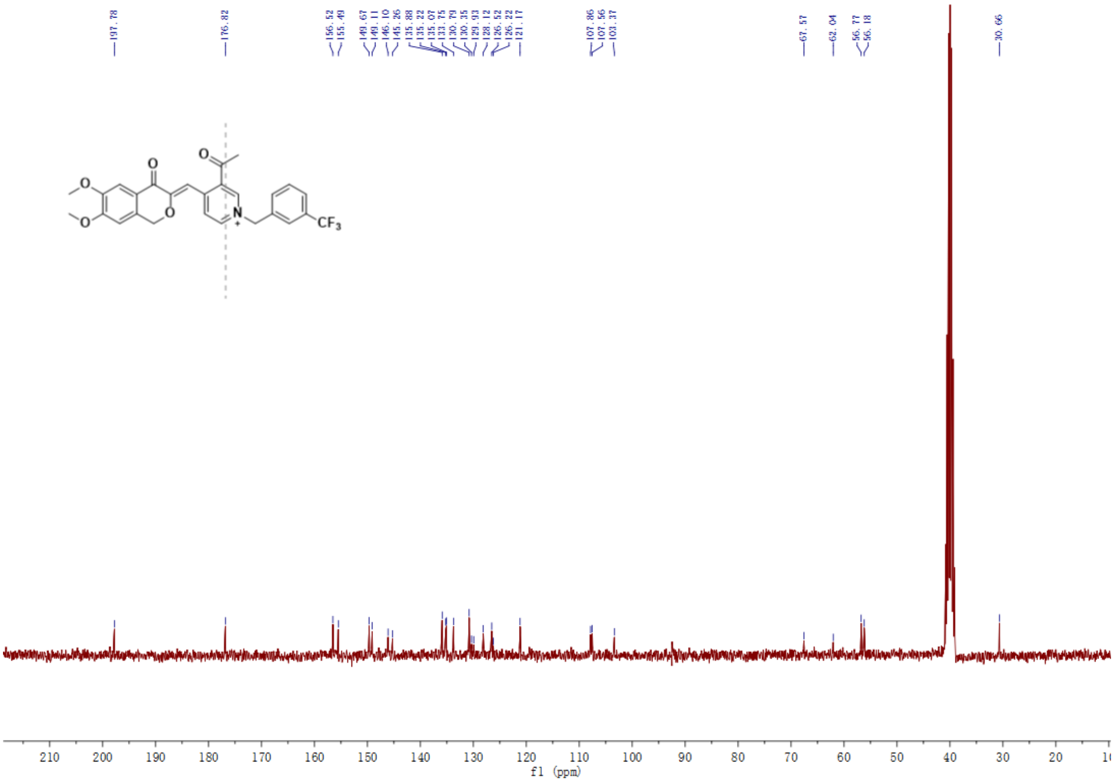

^{1}H-NMR and ^{13}C-HMR spectra of compound **10s**

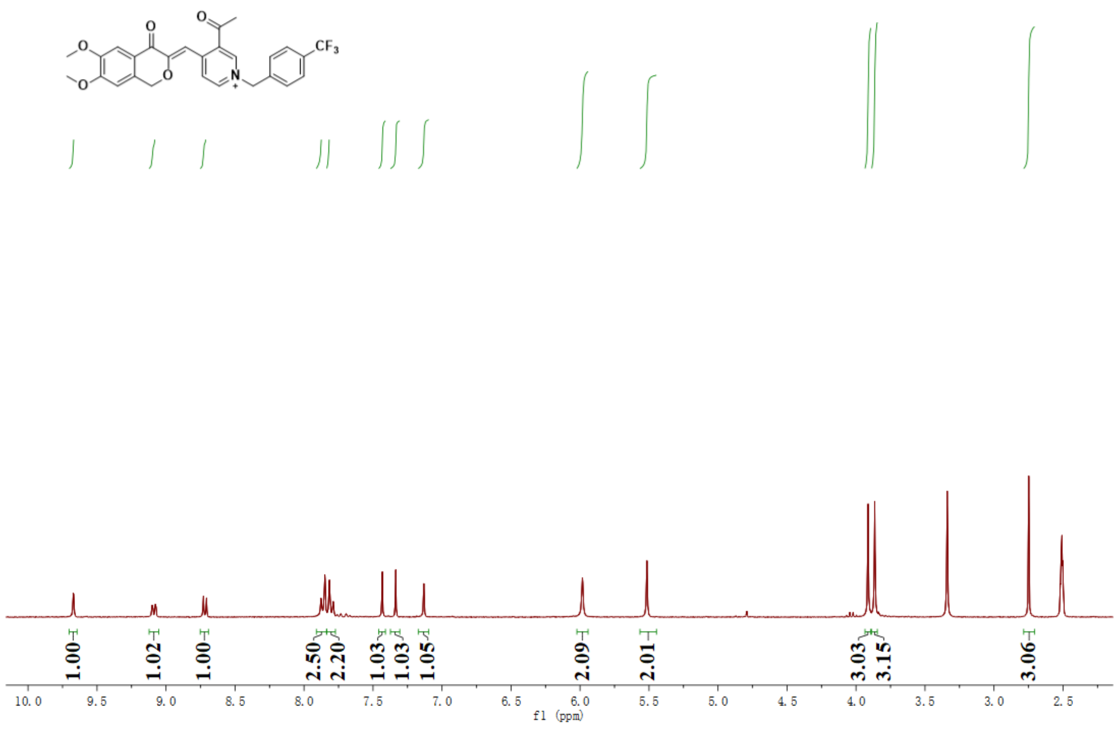

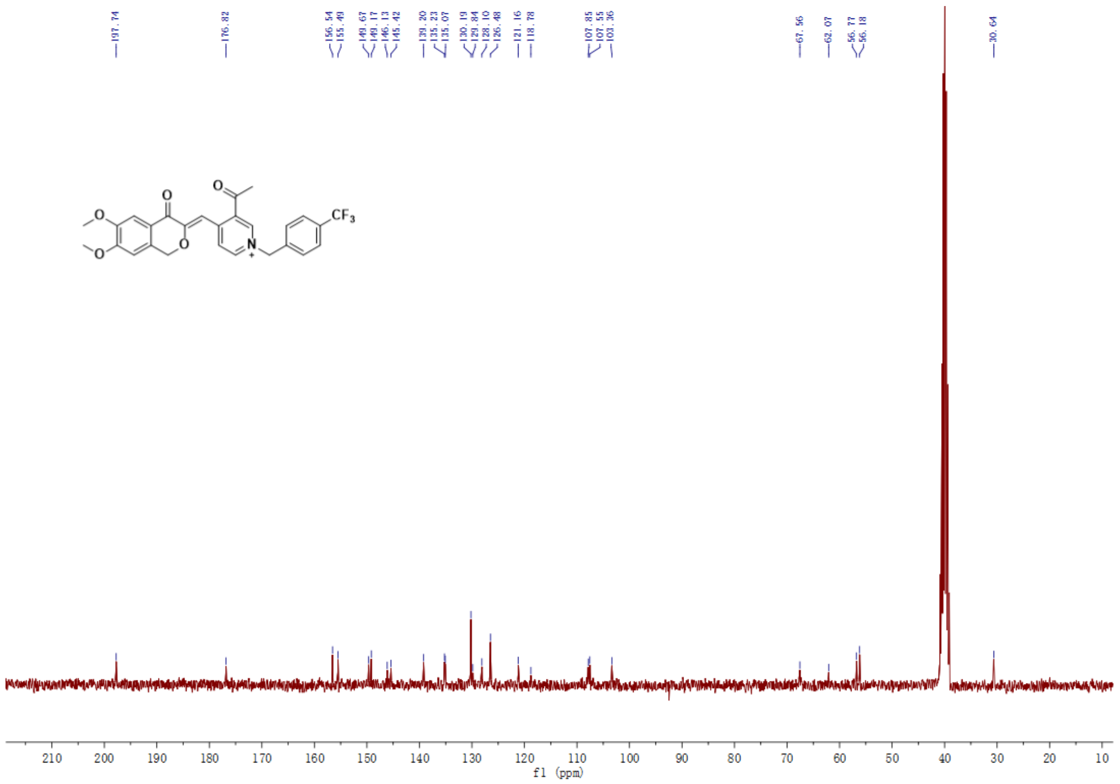

^{1}H-NMR and ^{13}C-HMR spectra of compound **13a**

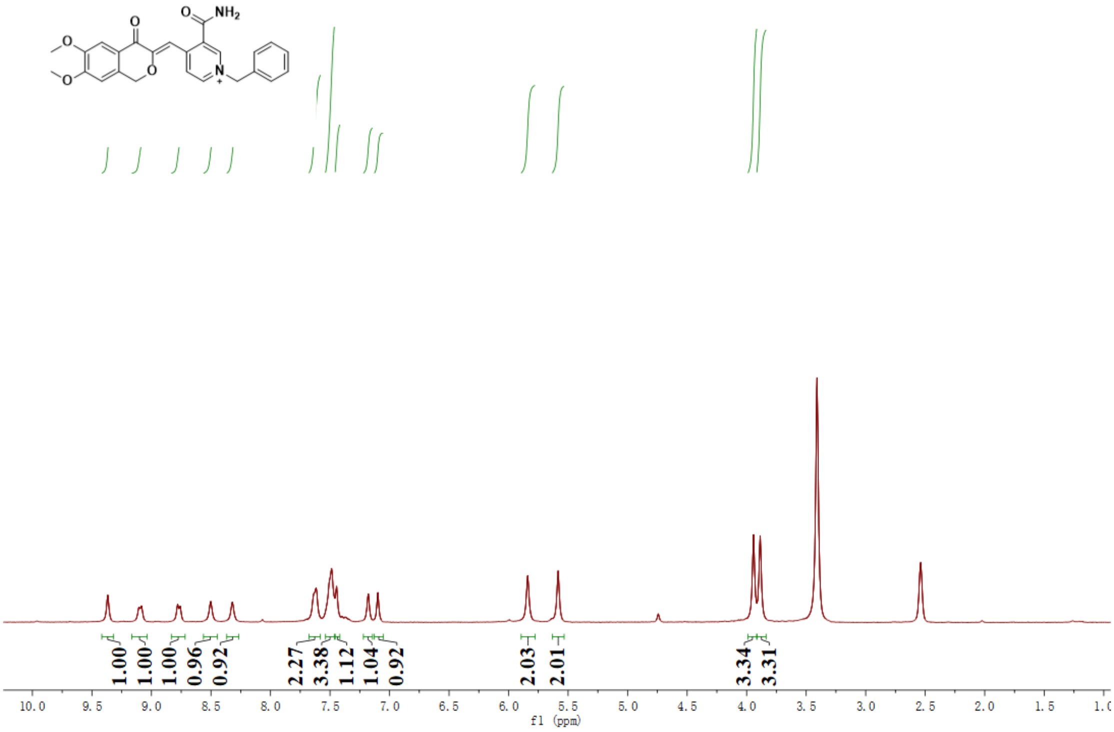

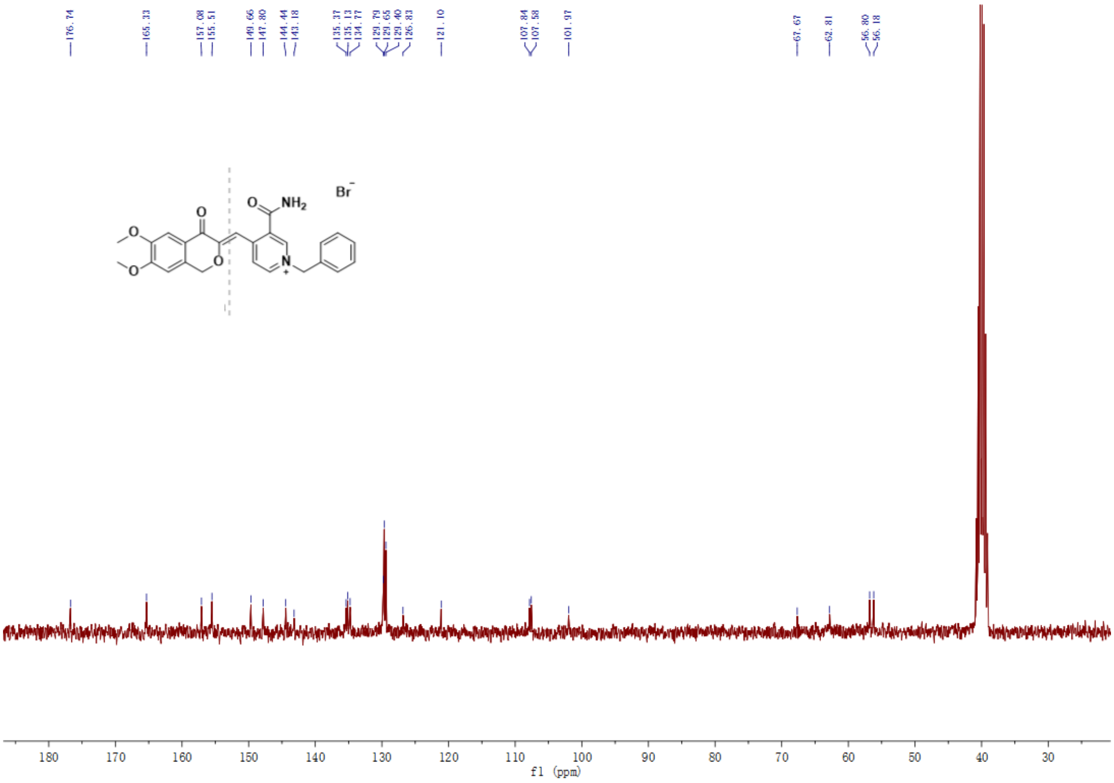

^{1}H-NMR and ^{13}C-HMR spectra of compound **13b**

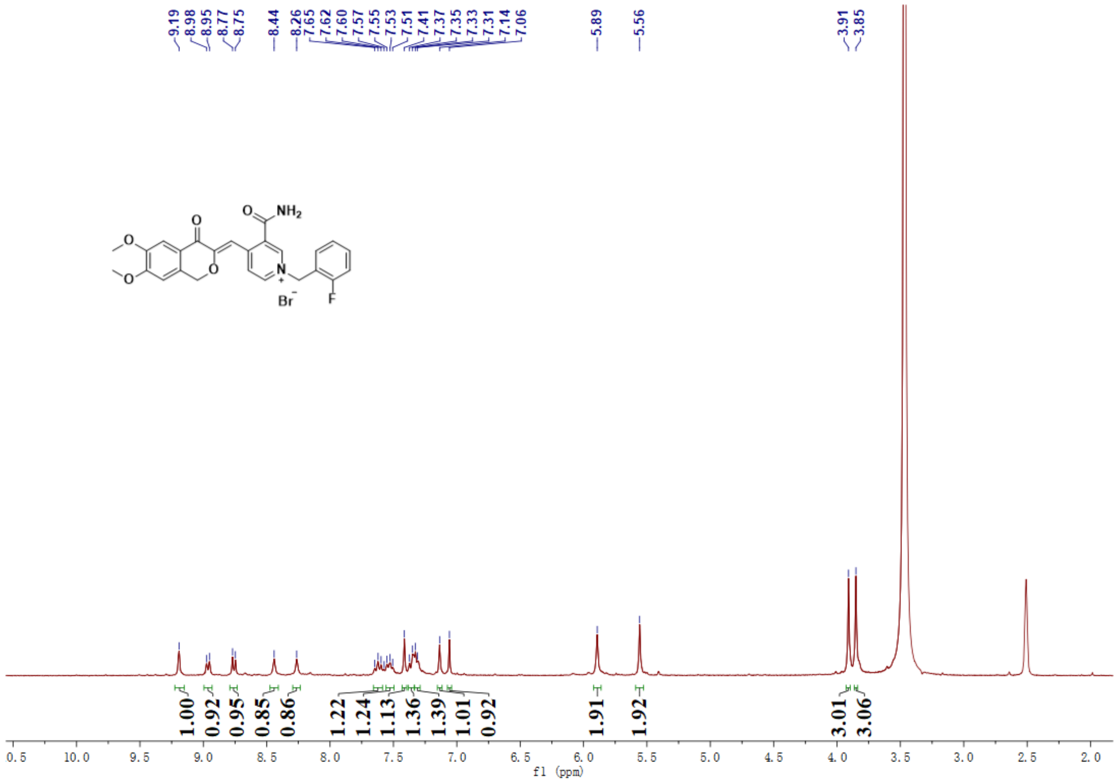

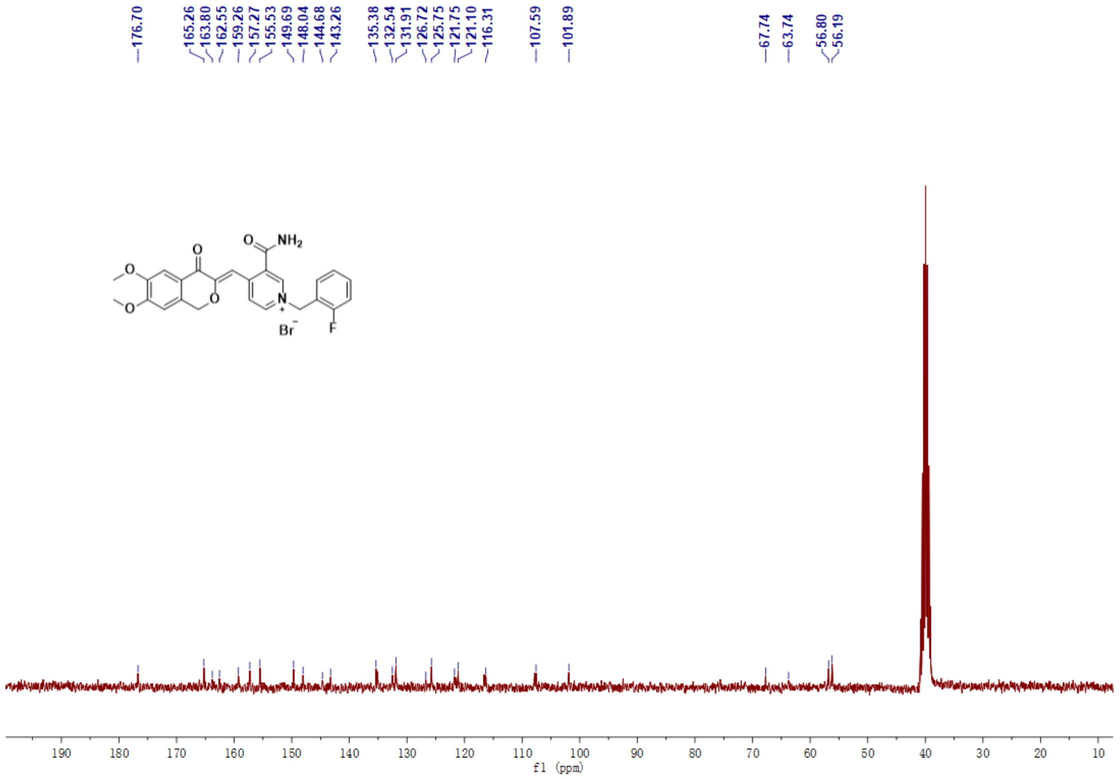

^{1}H-NMR and ^{13}C-HMR spectra of compound **13c**

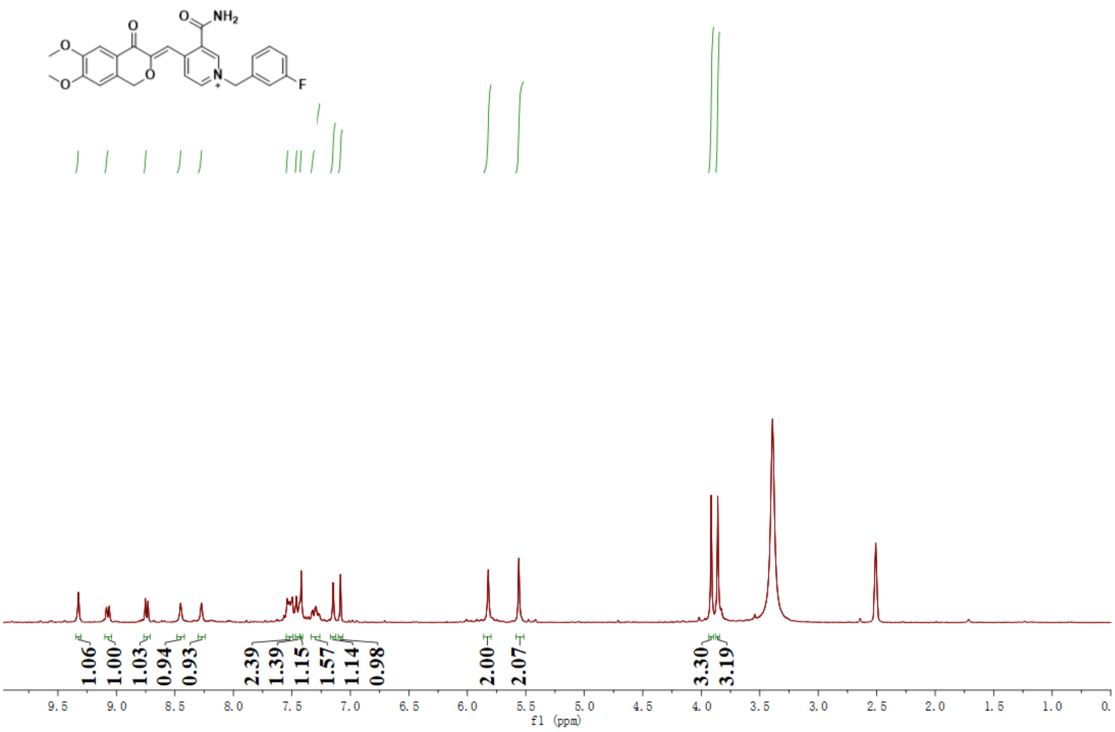

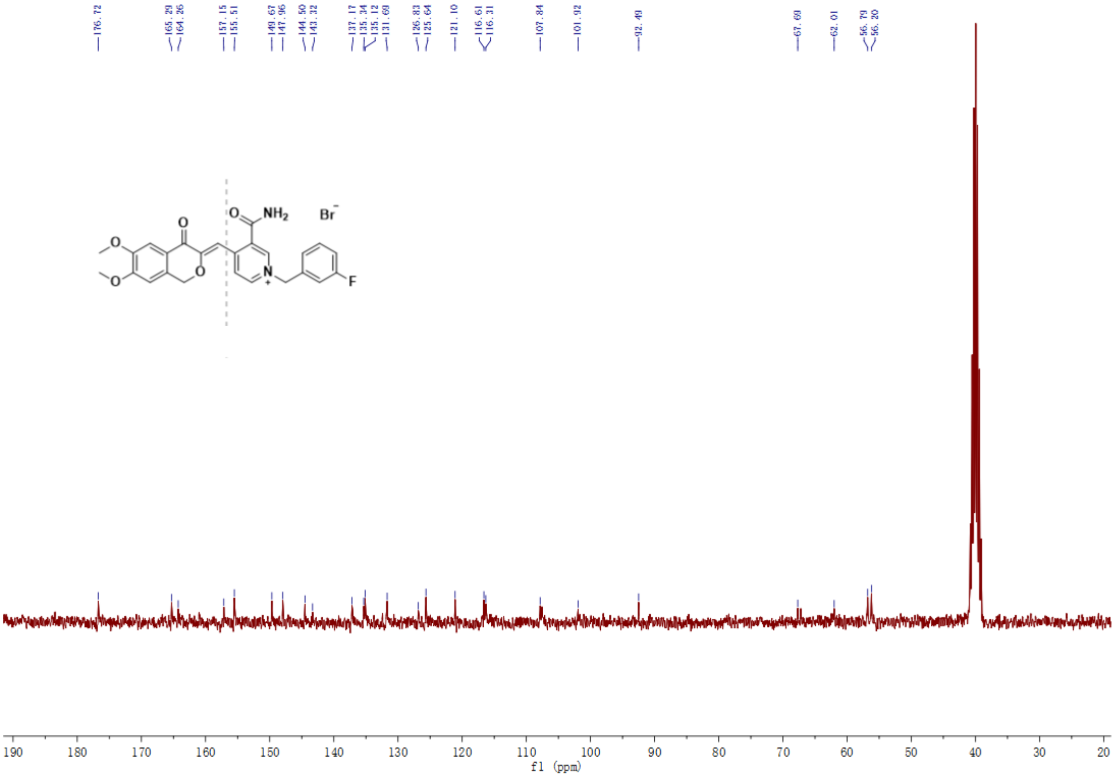

^{1}H-NMR and ^{13}C-HMR spectra of compound **13d**

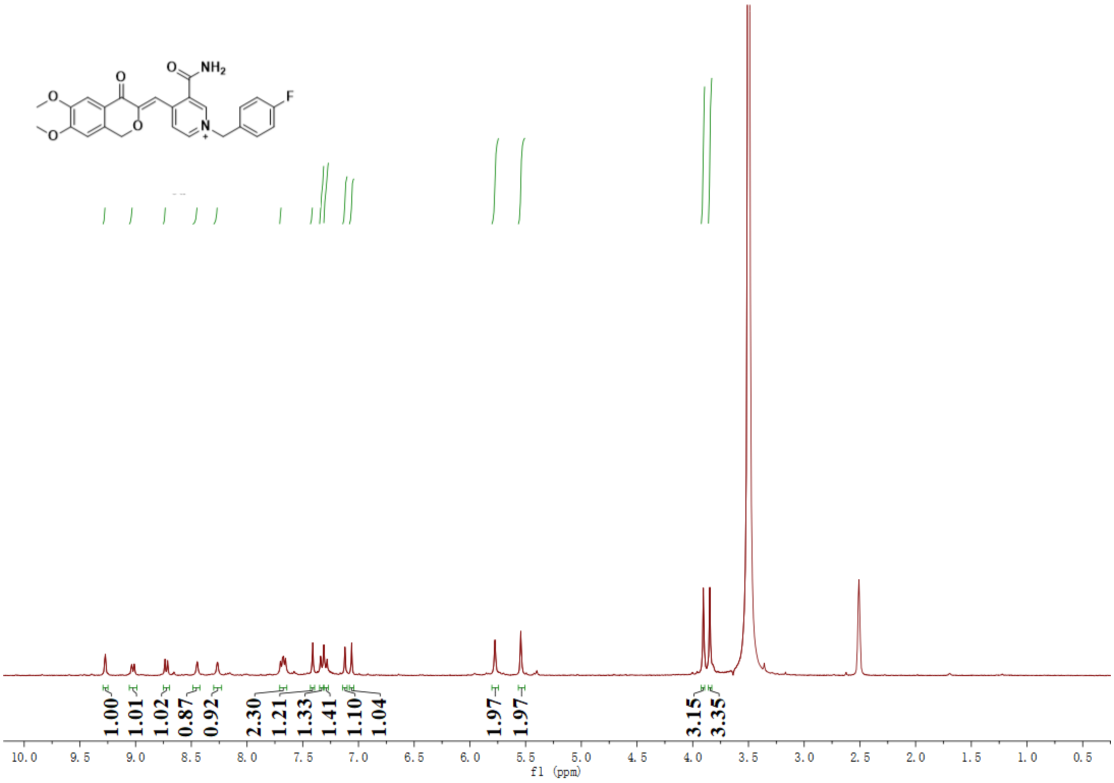

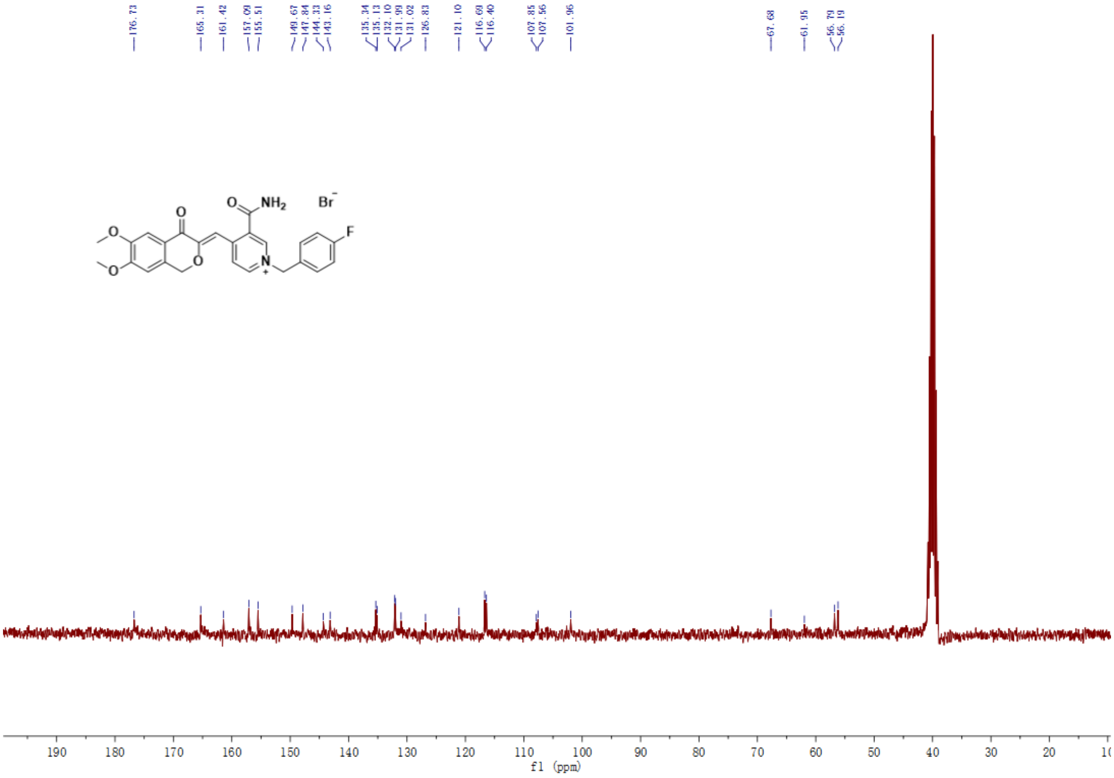

^{1}H-NMR and ^{13}C-HMR spectra of compound **13e**

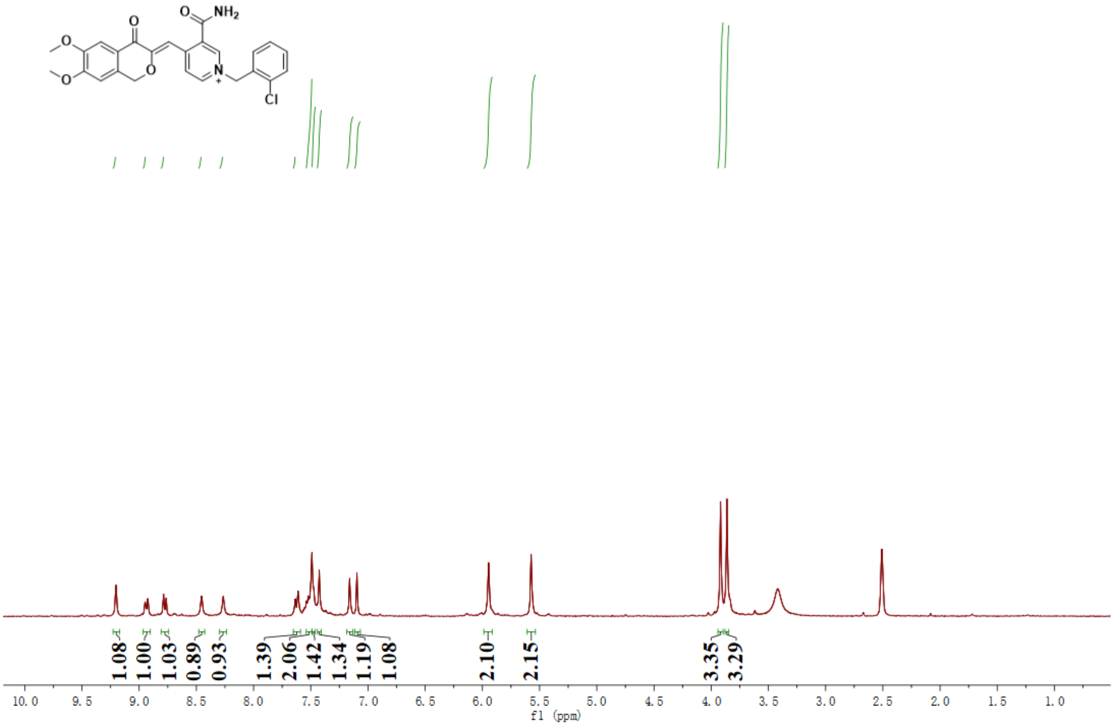

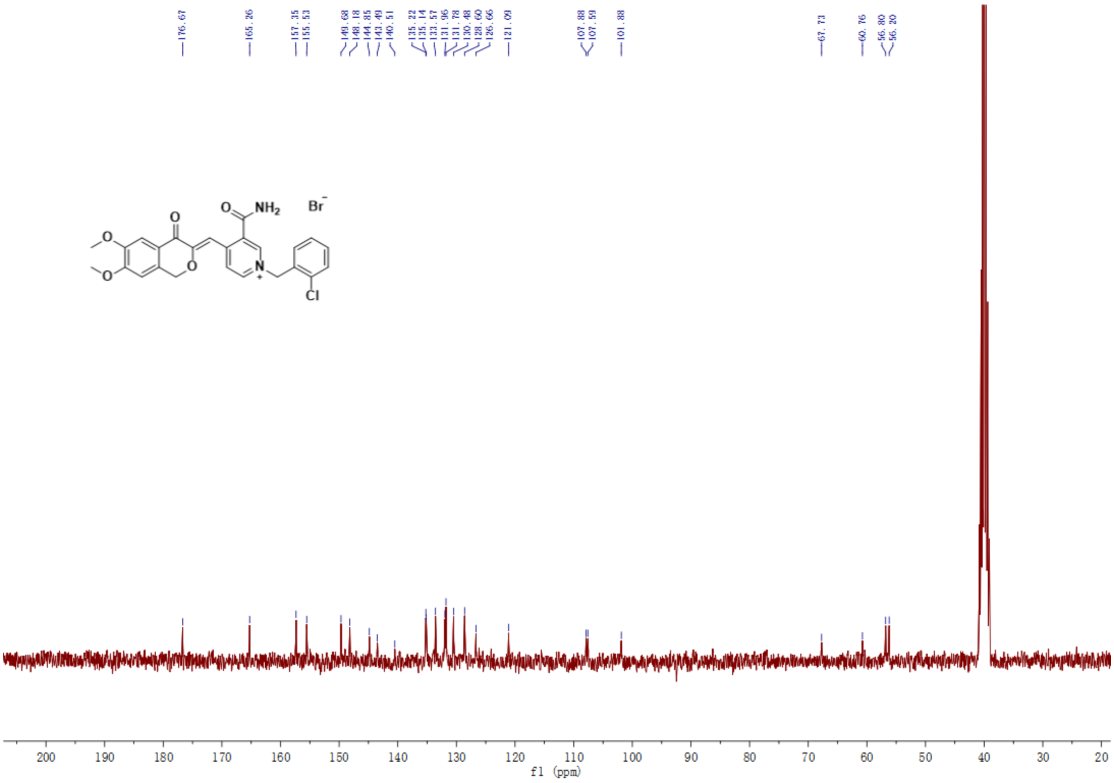

^{1}H-NMR and ^{13}C-HMR spectra of compound **13f**

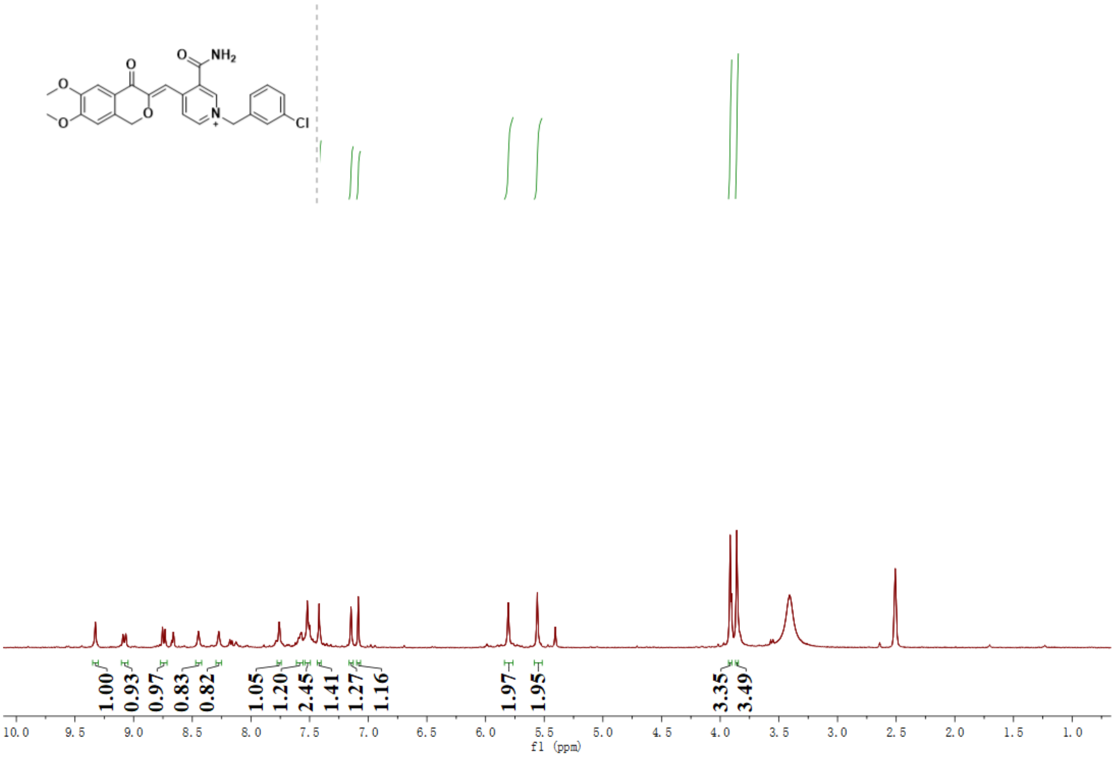

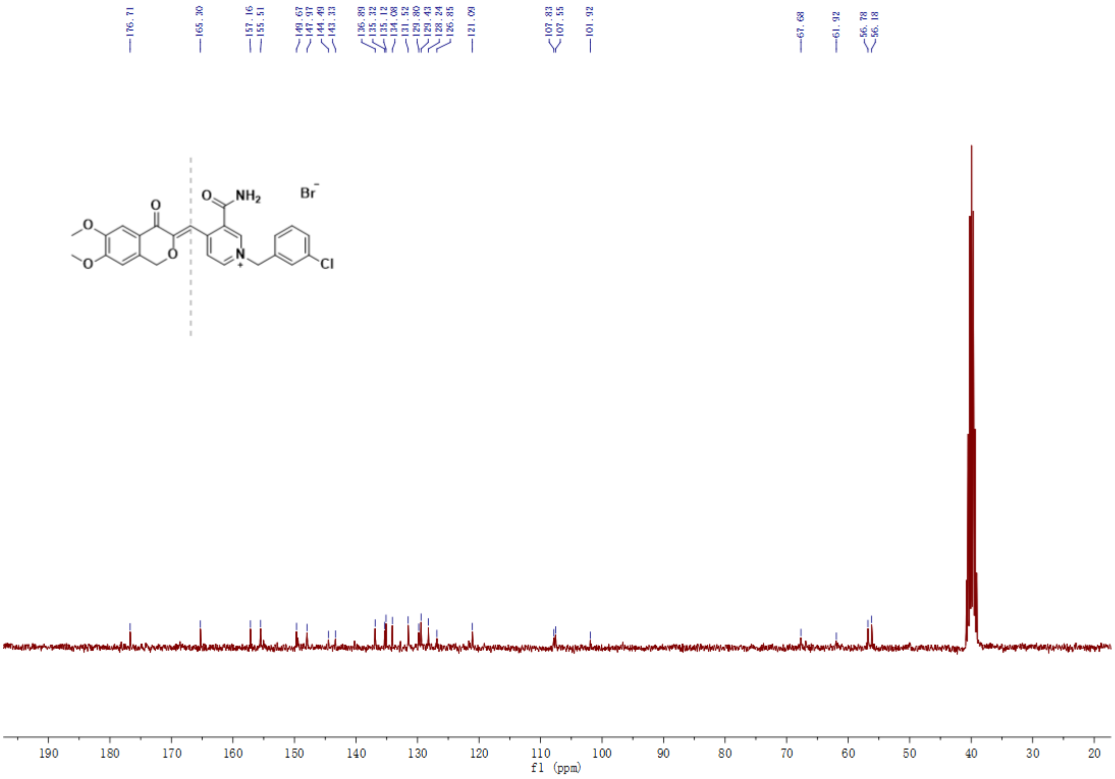

^{1}H-NMR and ^{13}C-HMR spectra of compound **13g**

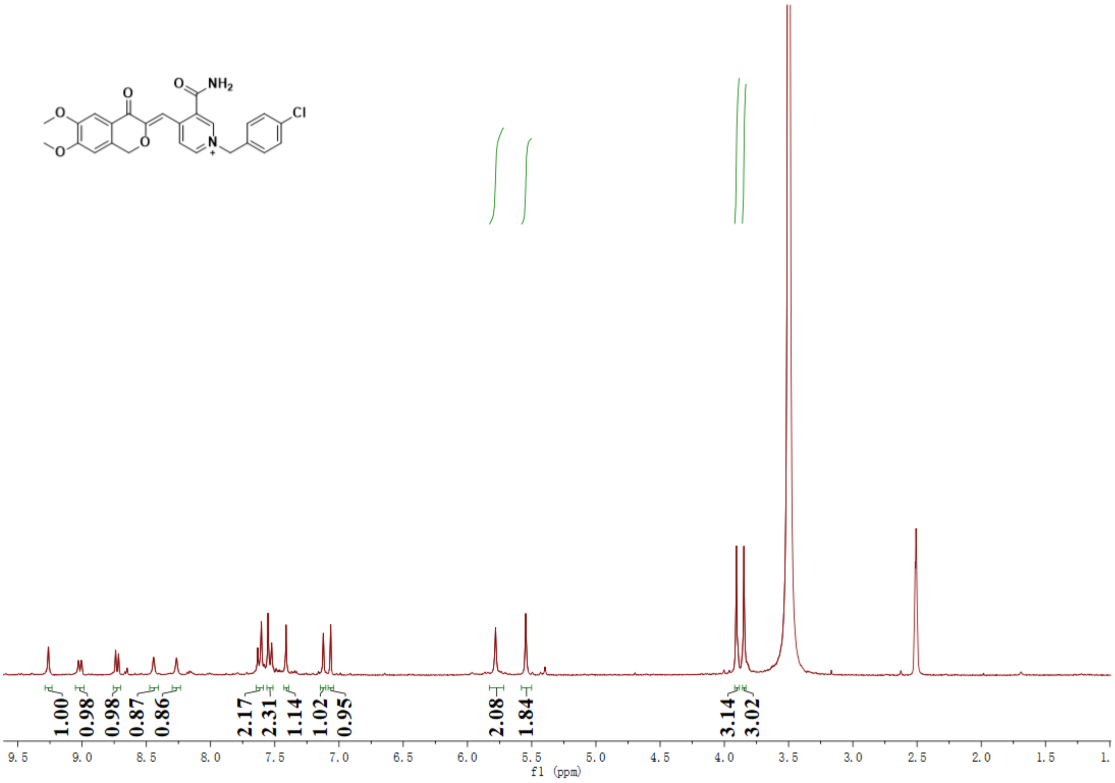

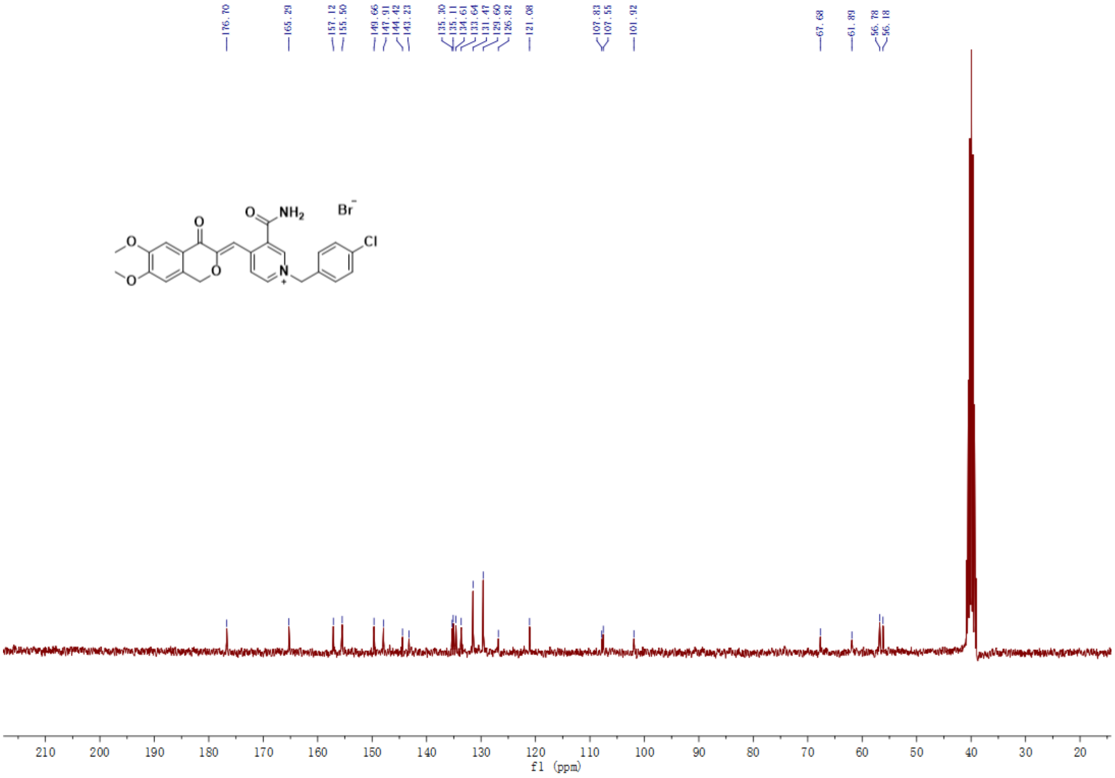

^{1}H-NMR and ^{13}C-HMR spectra of compound **13h**

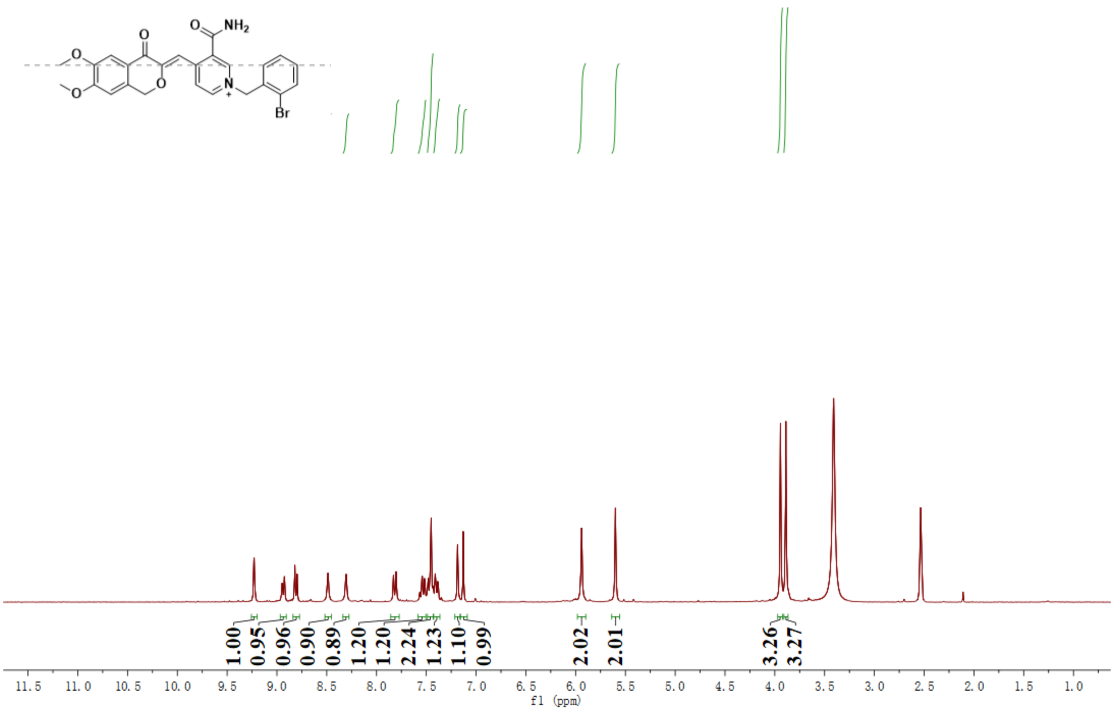

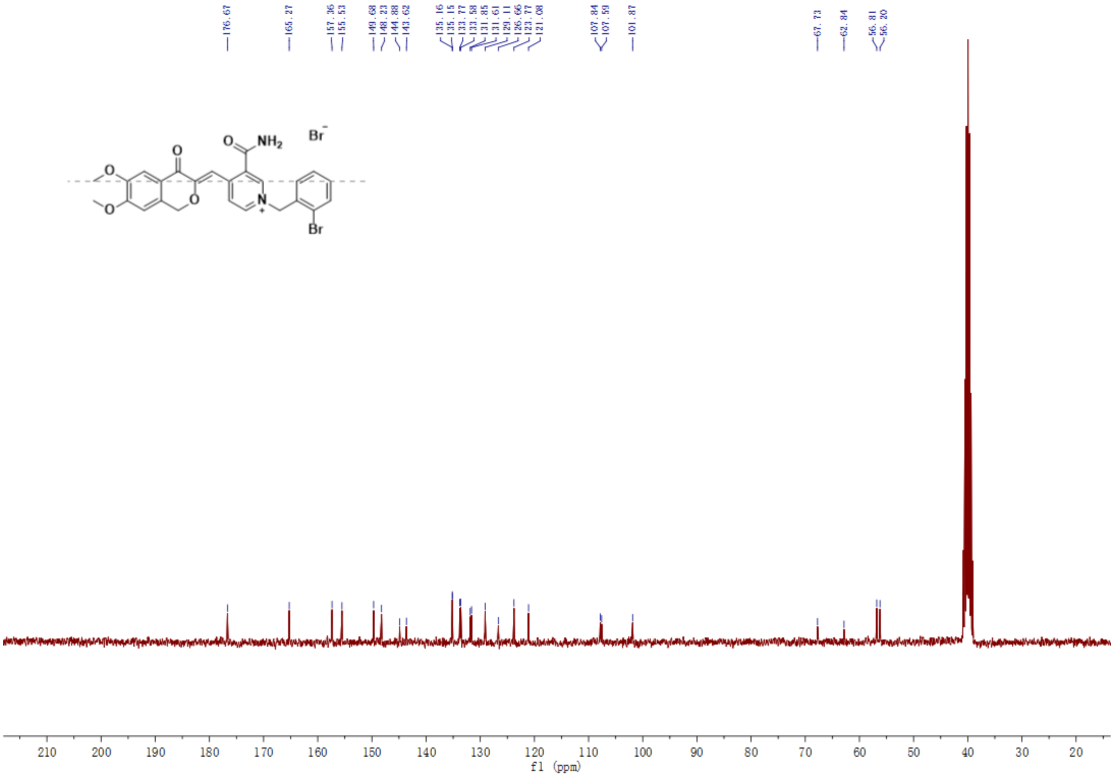

^{1}H-NMR and ^{13}C-HMR spectra of compound **13i**

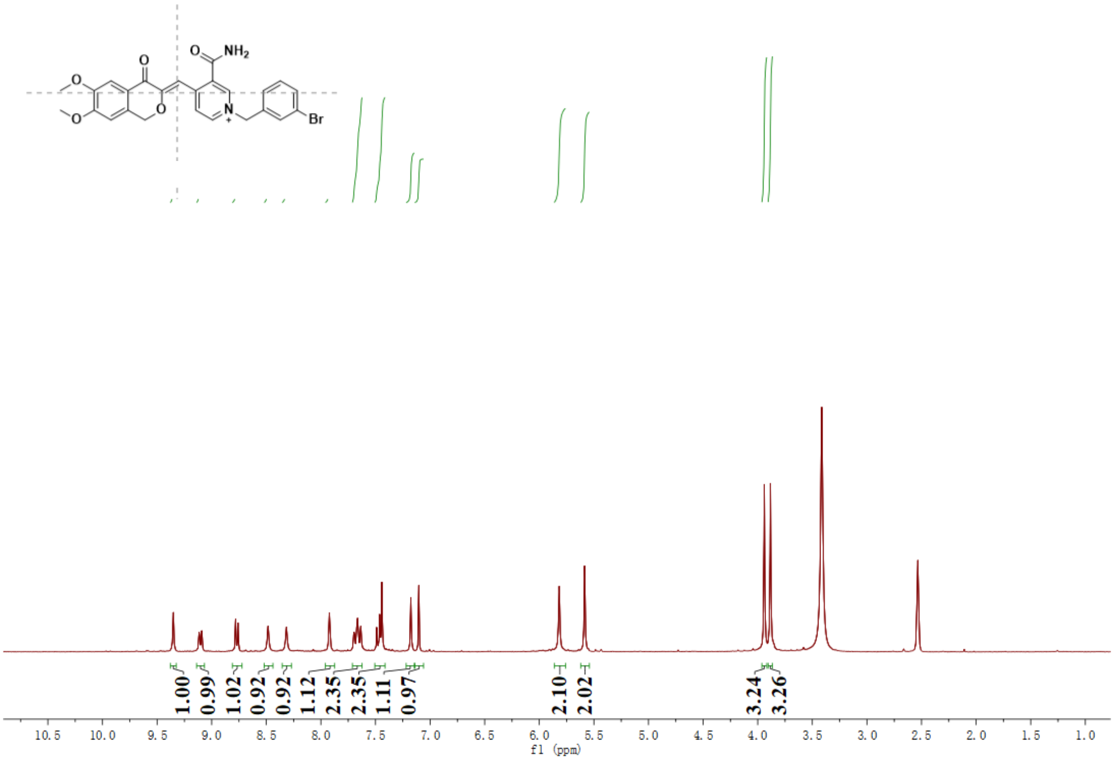

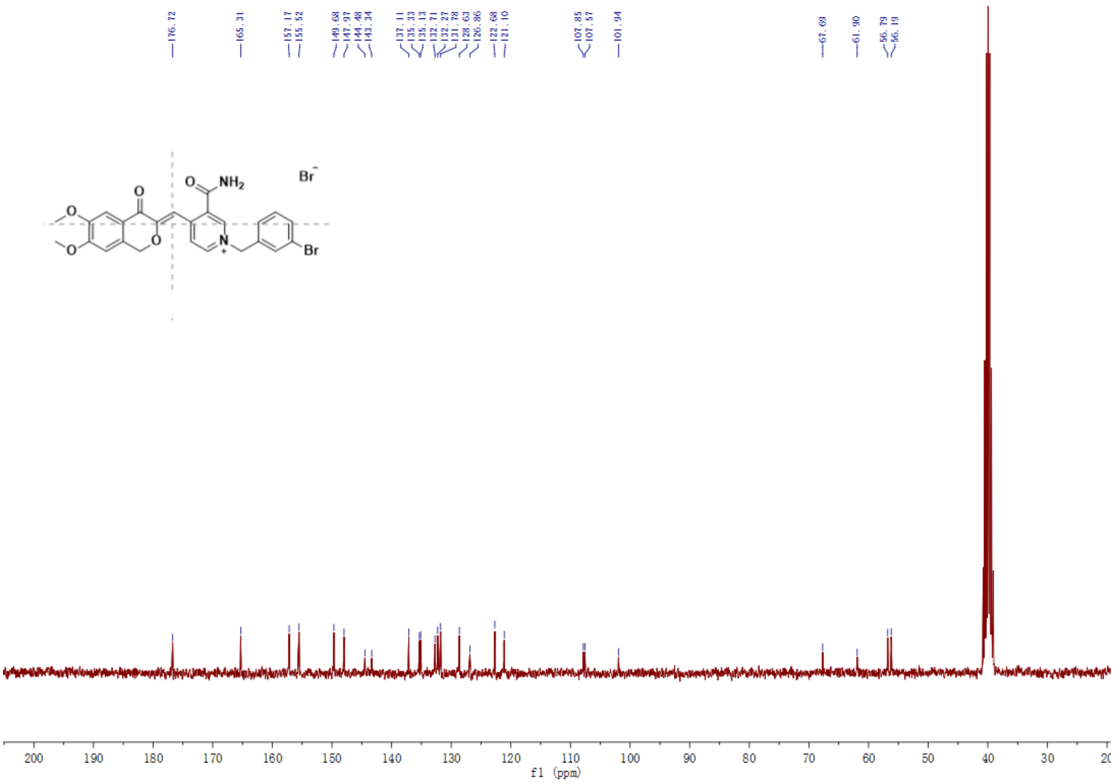

^{1}H-NMR and ^{13}C-HMR spectra of compound **13j**

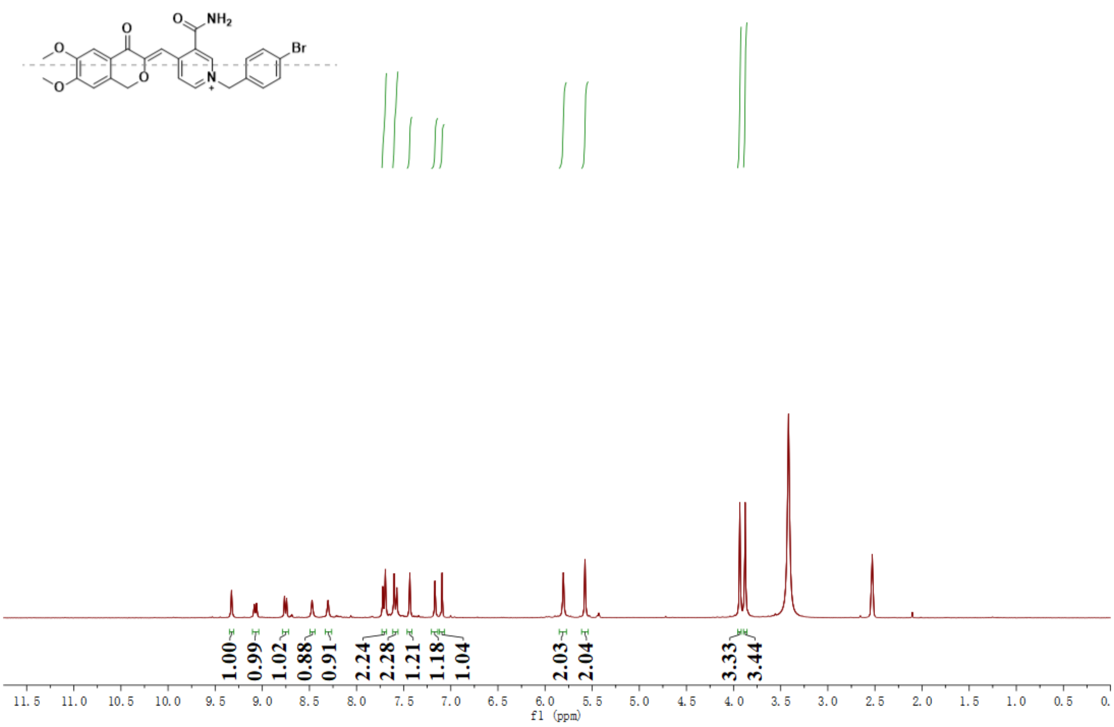

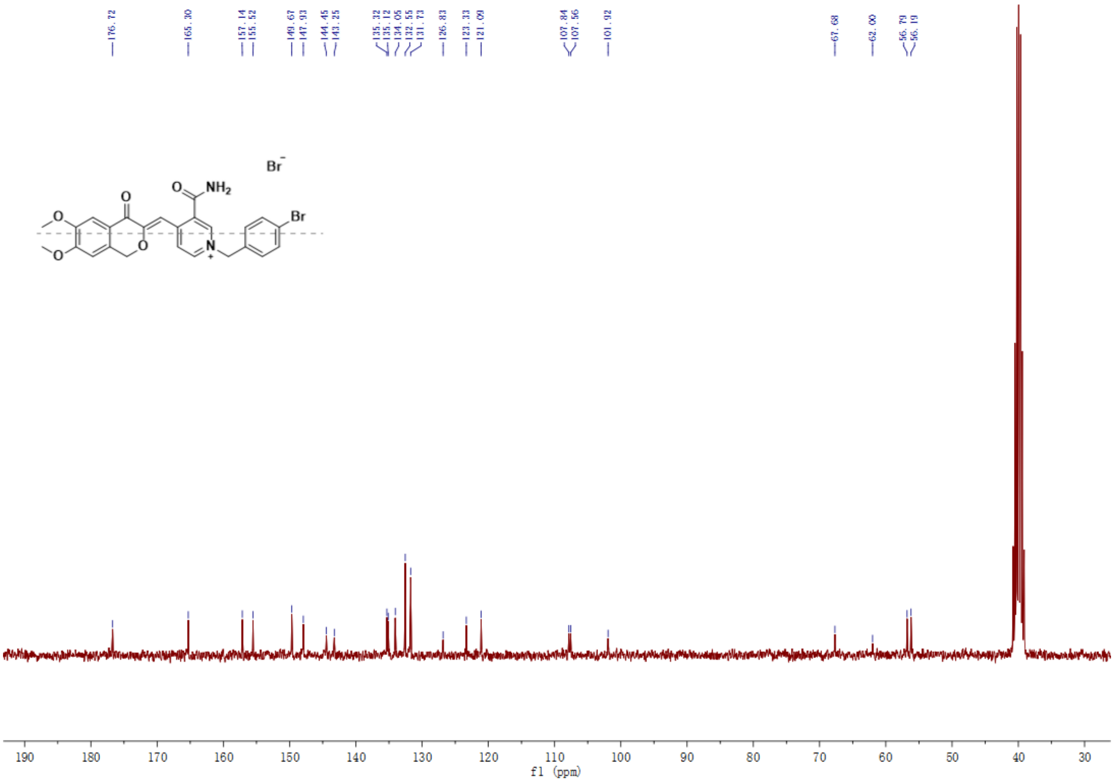

^{1}H-NMR and ^{13}C-HMR spectra of compound **13k**

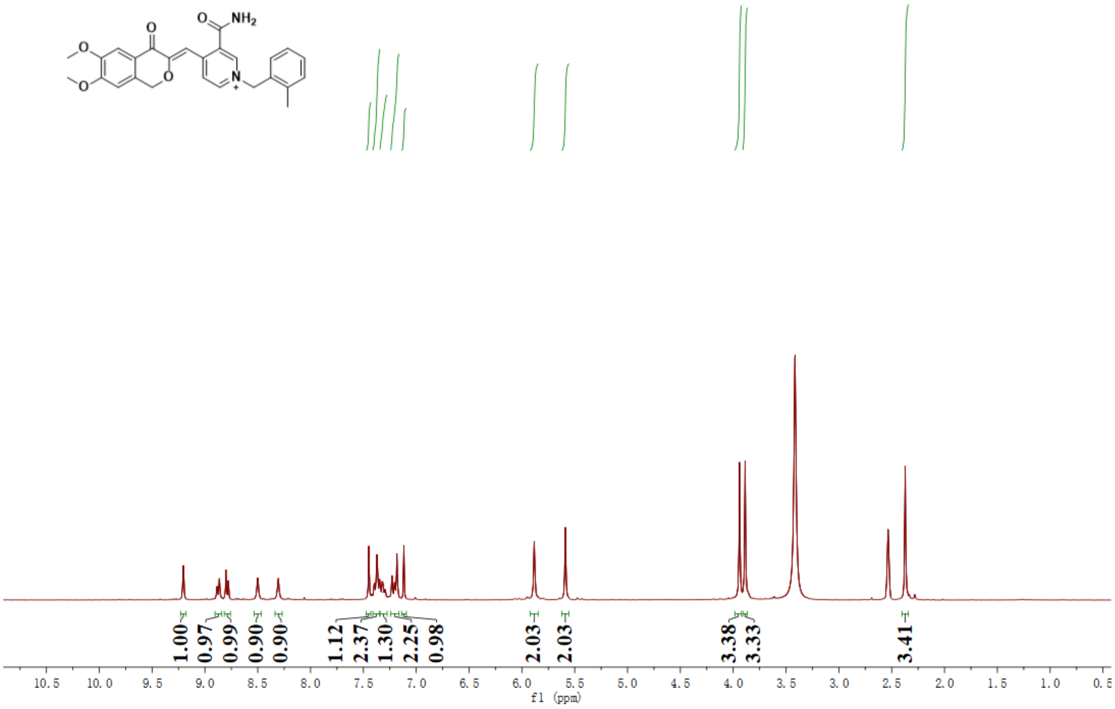

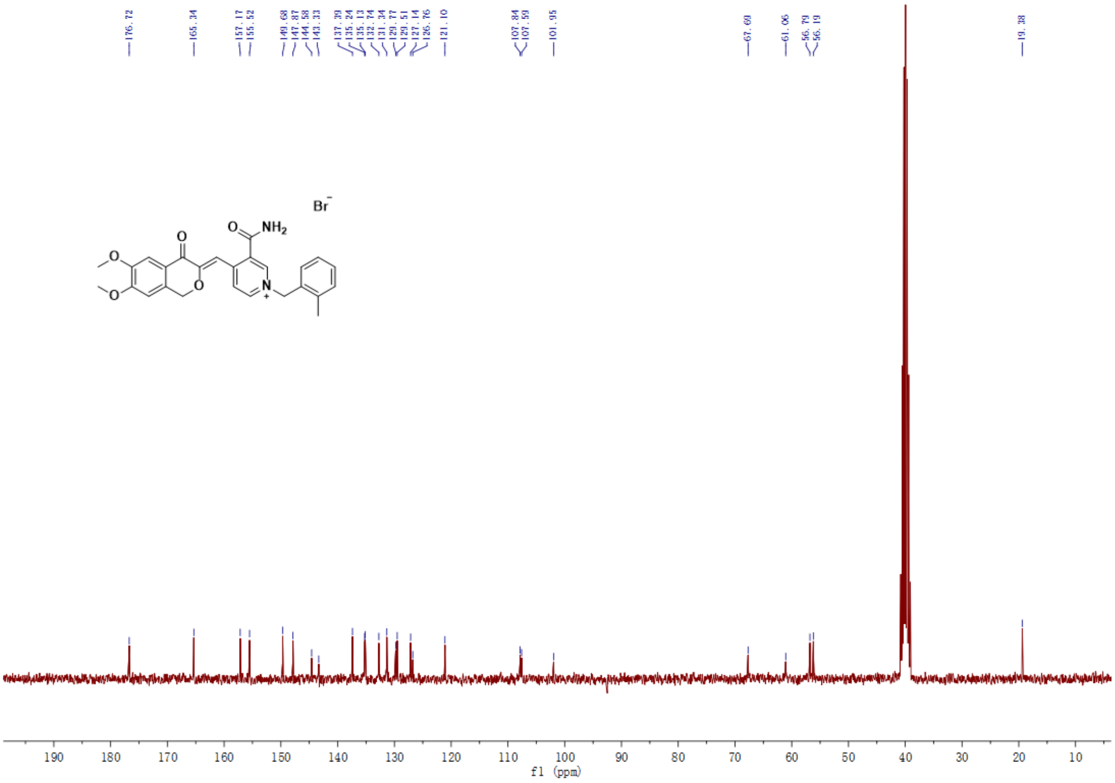

^{1}H-NMR and ^{13}C-HMR spectra of compound **13l**

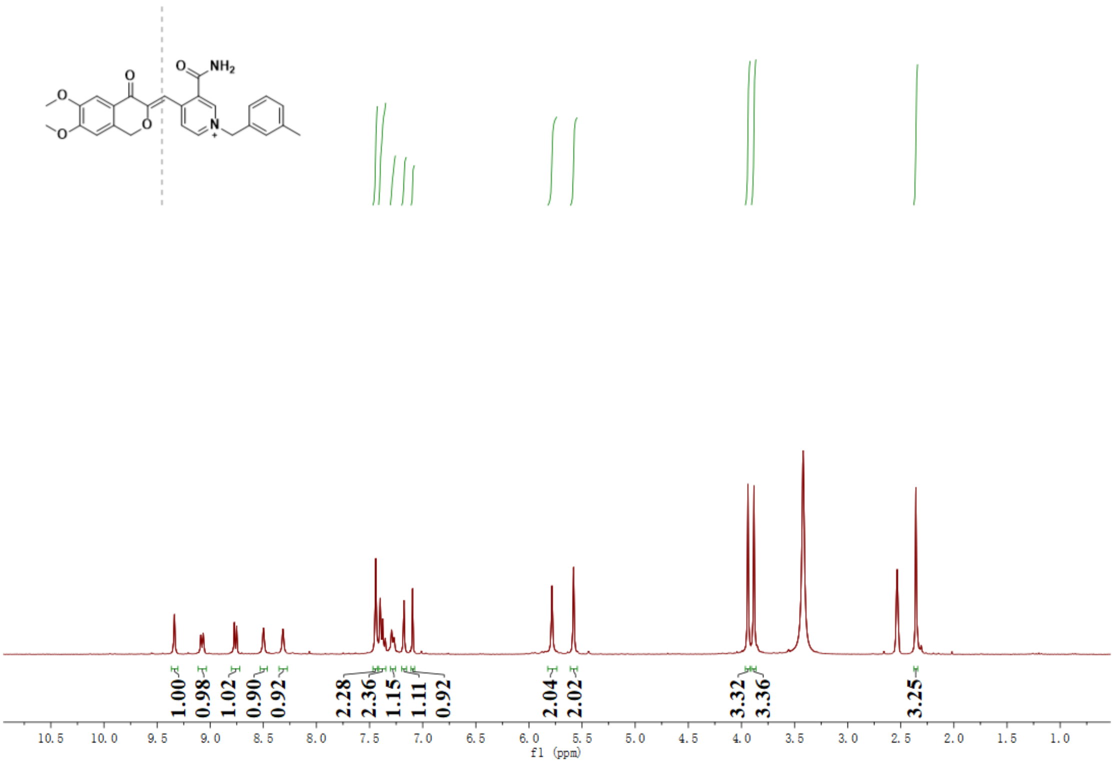

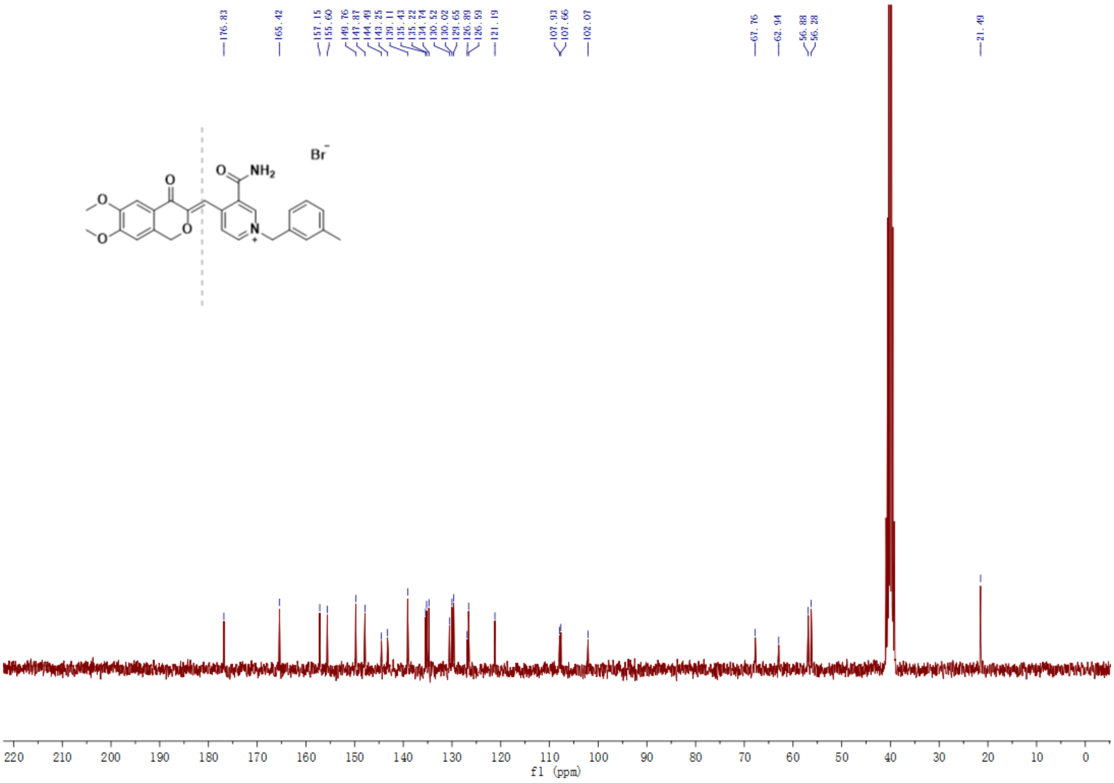

^{1}H-NMR and ^{13}C-HMR spectra of compound **13m**

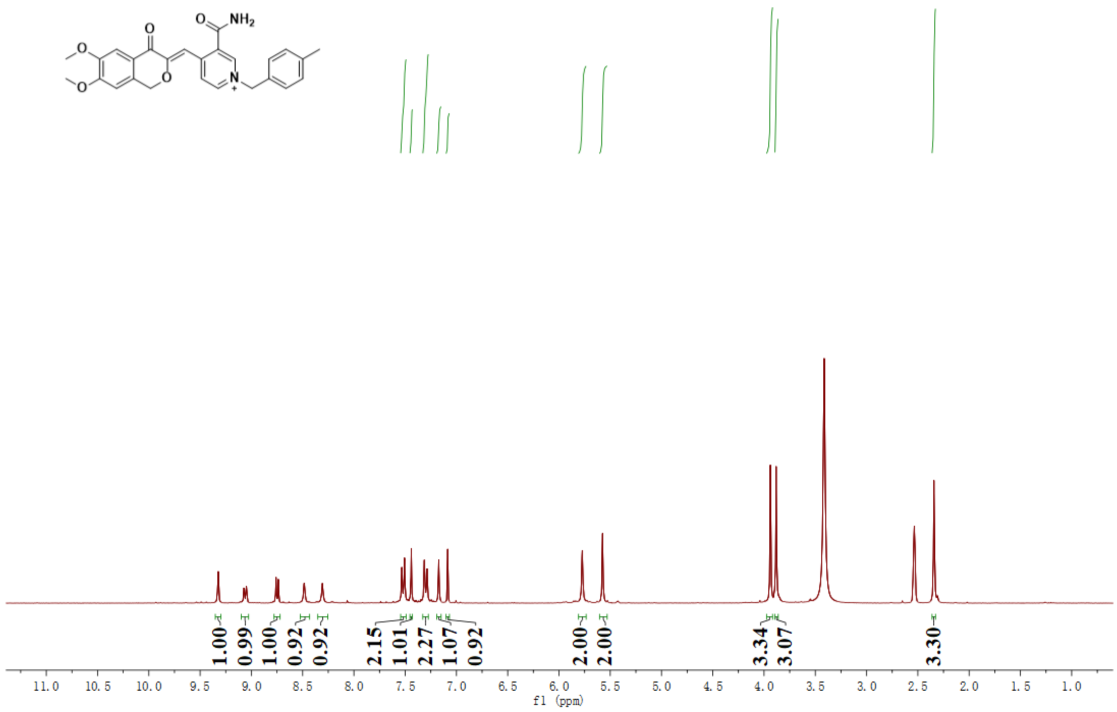

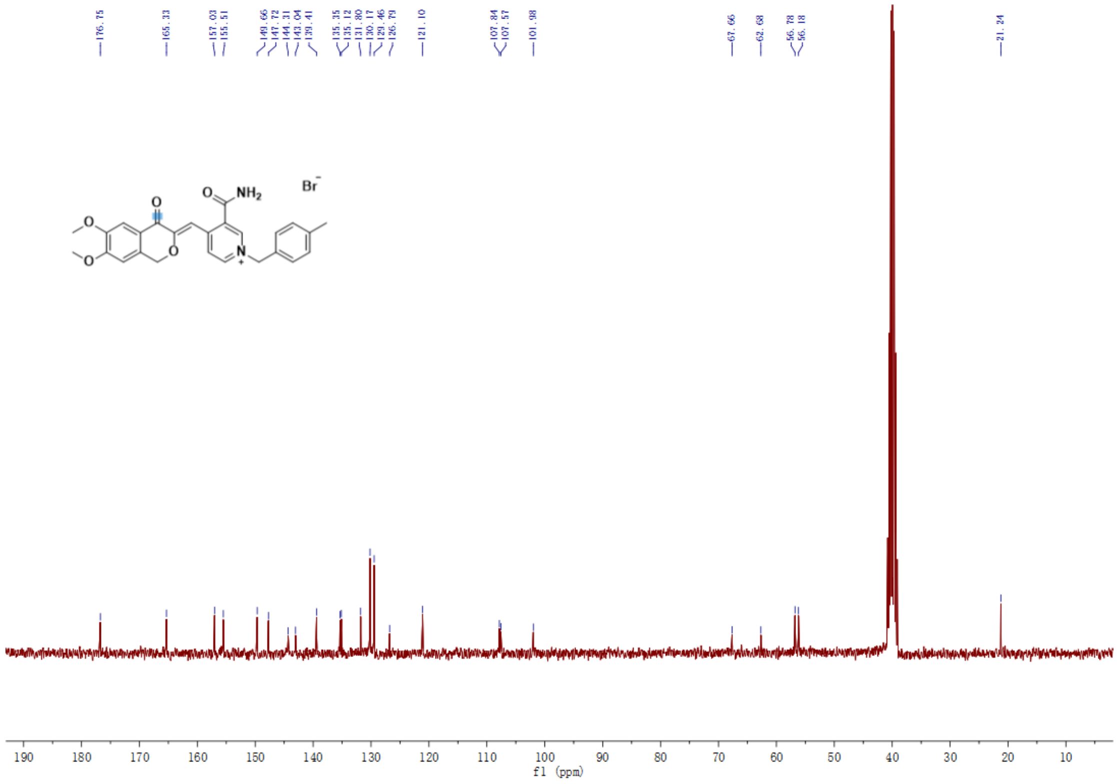

^{1}H-NMR and ^{13}C-HMR spectra of compound **13n**

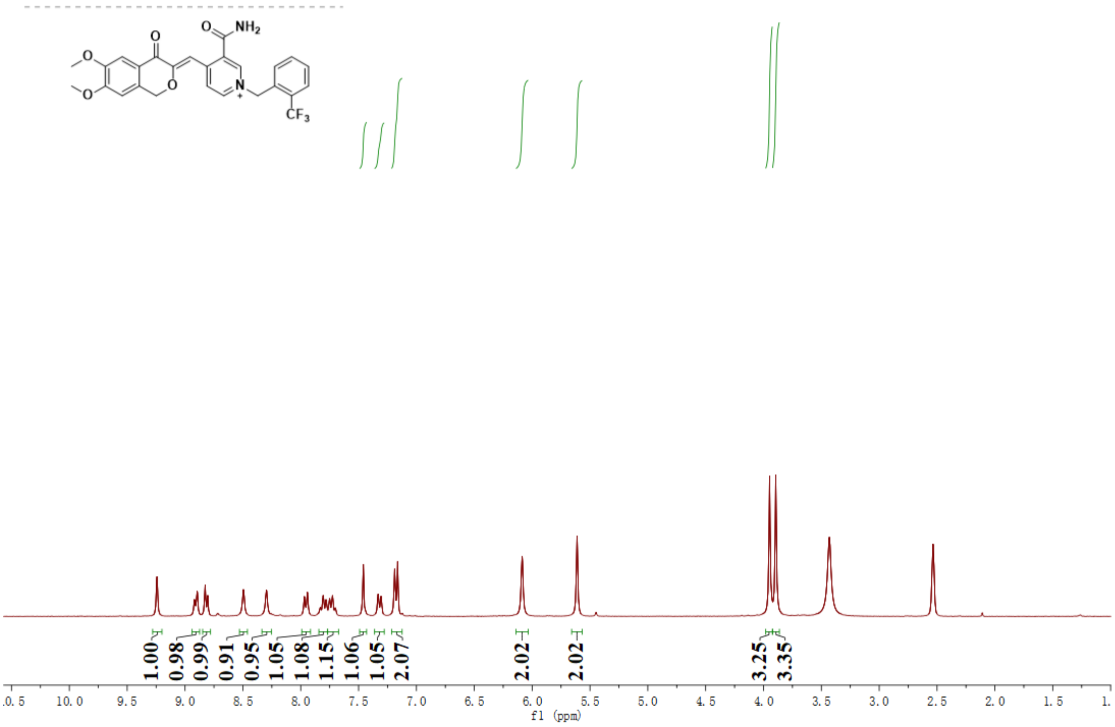

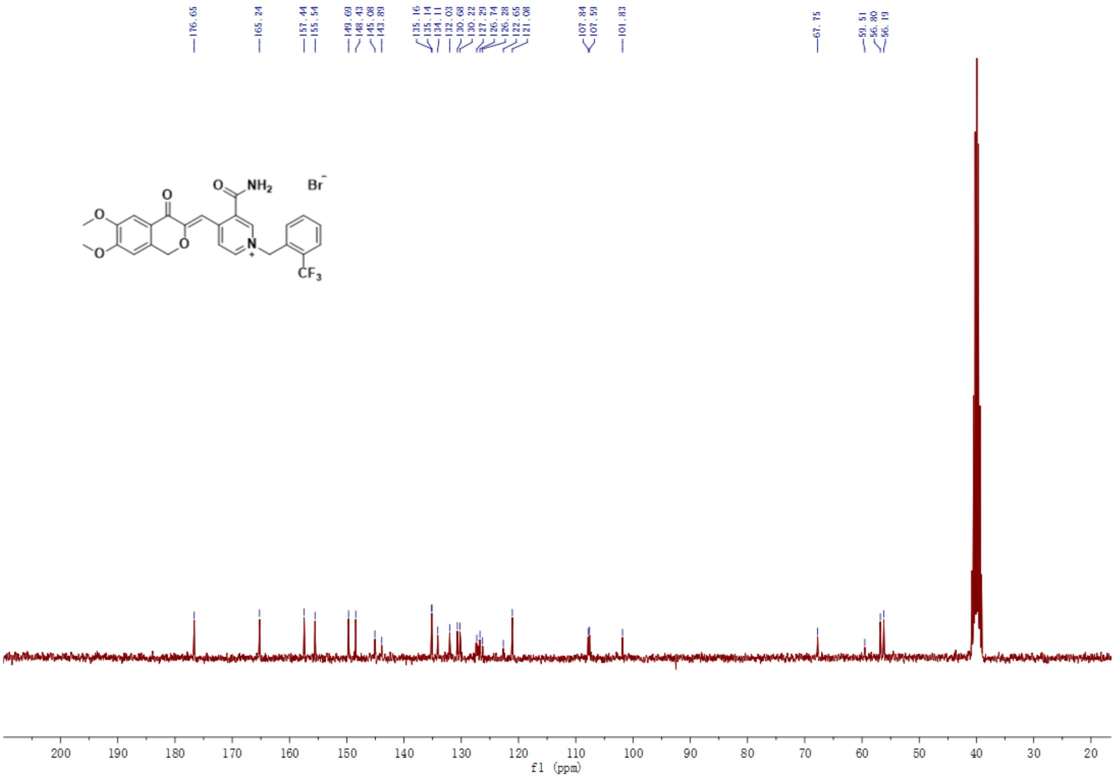

^{1}H-NMR and ^{13}C-HMR spectra of compound **13o**

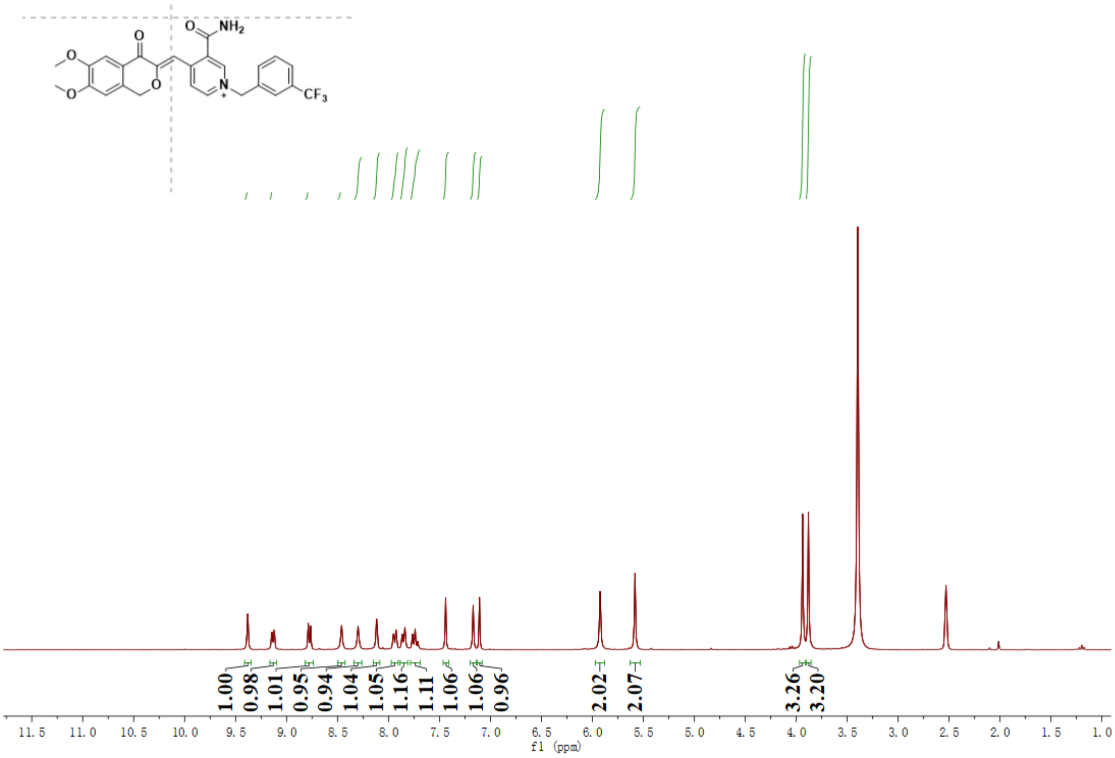

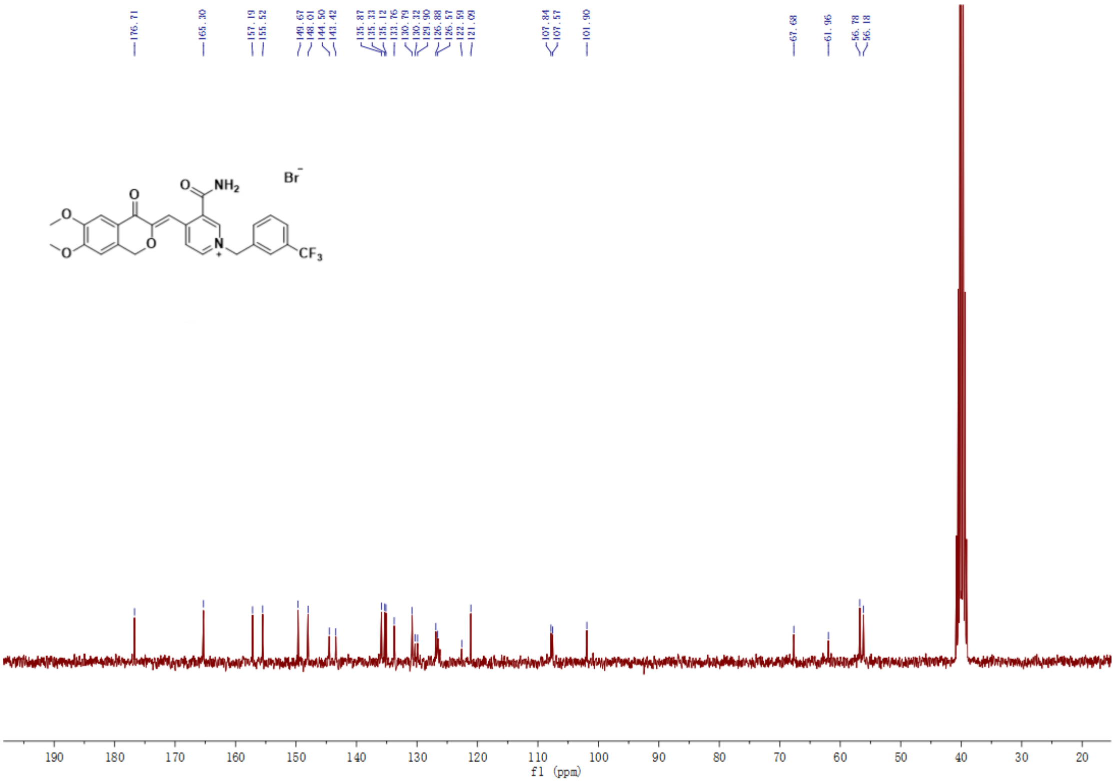

^{1}H-NMR and ^{13}C-HMR spectra of compound **13p**

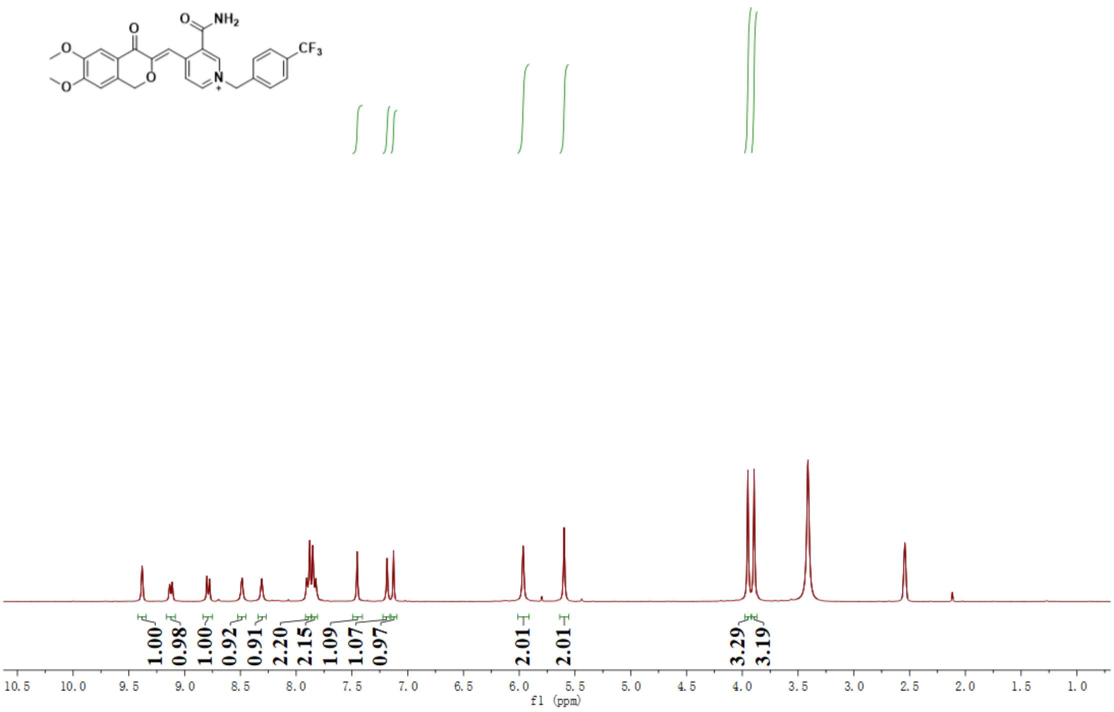

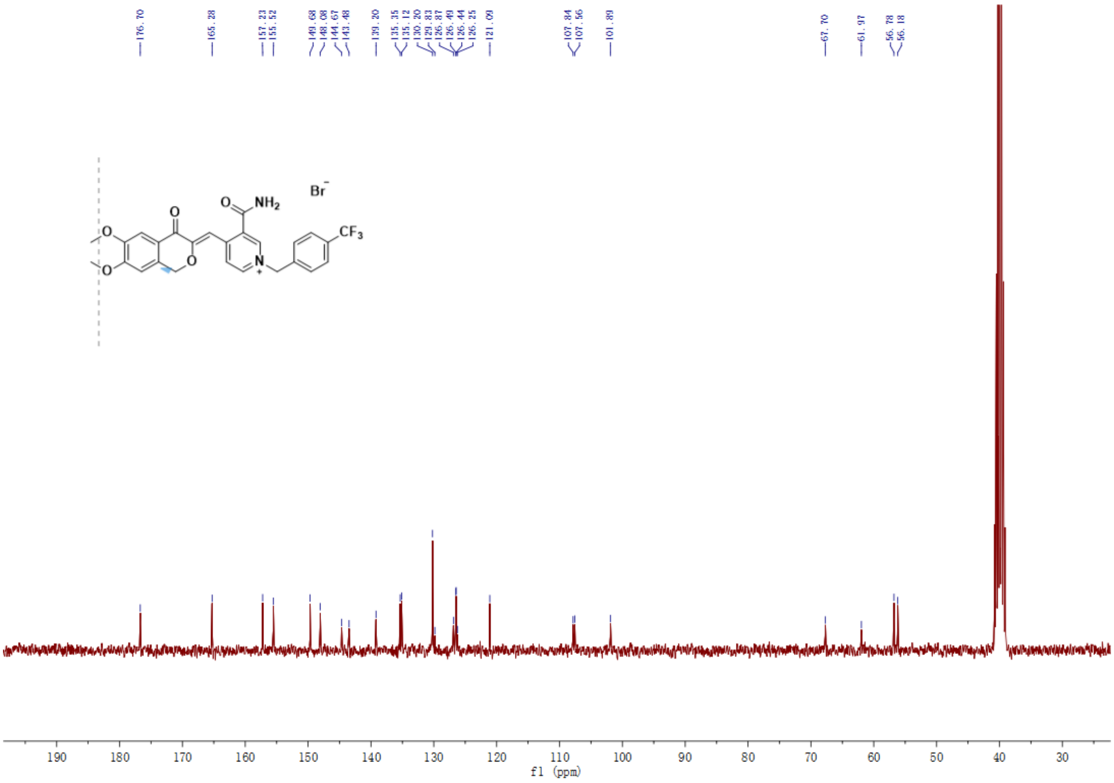

Supplement: Supplementary file 1 [file molecules-27-03090-s001.zip › molecules-1701302-supplementary.pdf]
